# The multiple functions and mechanisms of long non-coding RNAs in regulating breast cancer progression

**DOI:** 10.3389/fphar.2025.1559408

**Published:** 2025-03-28

**Authors:** Yongsheng Zhang, Yanjiao Xu, Yanping Zhang, Shoushi Wang, Mingqiang Zhao

**Affiliations:** ^1^ Qingdao Medical College, Qingdao University, Qingdao, Shandong, China; ^2^ Department of Anesthesiology, The Second Affiliated Hospital of Harbin Medical University, Harbin, Heilongjiang, China; ^3^ Department of Anesthesia and Perioperative Medicine, Qingdao Central Hospital, University of Health and Rehabilitation Sciences, Qingdao, Shandong, China

**Keywords:** lncRNAs, BC, cell cycle, the tumor microenvironment, autophagy, drug resistance

## Abstract

Breast cancer (BC) is a malignant tumor that has the highest morbidity and mortality rates in the female population, and its high tendency to metastasize is the main cause of poor clinical prognosis. Long non-coding RNAs (lncRNAs) have been extensively documented to exhibit aberrant expression in various cancers and influence tumor progression via multiple molecular pathways. These lncRNAs not only modulate numerous aspects of gene expression in cancer cells, such as transcription, translation, and post-translational modifications, but also play a crucial role in the reprogramming of energy metabolism by regulating metabolic regulators, which is particularly significant in advanced BC. This review examines the characteristics and mechanisms of lncRNAs in regulating BC cells, both intracellularly (e.g., cell cycle, autophagy) and extracellularly (e.g., tumor microenvironment). Furthermore, we explore the potential of specific lncRNAs and their regulatory factors as molecular markers and therapeutic targets. Lastly, we summarize the application of lncRNAs in the treatment of advanced BC, aiming to offer novel personalized therapeutic options for patients.

## 1 Introduction

BC is the second most prevalent cancer globally, following lung cancer, and it is the most frequently diagnosed cancer among women, constituting one of the principal causes of cancer-related mortality (Bray et al., 2024). In recent years, significant strides have been made to improve BC treatment. Conventional therapeutic approaches encompass local resection, radical surgery, endocrine therapy, targeted therapy, chemoradiotherapy, and immunotherapy (Singh D. et al., 2022). Early diagnosis and personalized treatment strategies have substantially increased the lifespan of BC patients, with the 5-year survival rate for early-stage BC approaching 100%. Nevertheless, organ damage resulting from metastasis significantly impairs treatment efficacy. The development of metastatic lesions in BC often signifies the loss of the optimal treatment window, indicating a poor prognosis. While chemotherapy, hormone therapy, and radiotherapy can delay tumor progression and modestly extend patient survival (Steeg, 2006). Approximately one-third of BC patients experience distant organ metastases, reducing the 5-year survival rate to 29% (Torre et al., 2015; Giaquinto et al., 2022). Nearly all BC-related deaths are attributed to tumor metastasis, as advanced metastatic cancer remains incurable (Yofe et al., 2023).

The metastasis of BC is recognized as a multifaceted biological phenomenon, potentially necessitating a prolonged incubation period. The timing, location, and degree of malignancy of metastasis vary significantly among individuals, and numerous questions regarding the interplay between the primary tumor and metastatic lesions remain unresolved (Nguyen et al., 2009). The initial site of metastasis is typically the lymph nodes, which is strongly correlated with adverse prognosis. Metastasis can extend beyond the mammary parenchyma via lymphatic and vascular routes, affecting other organs such as the kidneys, bones, brain, liver, or lungs (Schito and Rey, 2017). BC metastasis is marked by its complexity, heterogeneity, and genomic instability, and recent research has demonstrated that lncRNAs play a crucial role in this process (Marino et al., 2013; Li et al., 2016).

The majority of mammalian noncoding genomes are transcribed in a cell-specific manner, generating noncoding RNAs exceeding 200 nucleotides in length. Research has demonstrated that the expression of most lncRNAs is highly specific, with their expression in the adult mouse brain being associated with particular tissues, cell types, and subcellular localization, exhibiting a more precise expression profile compared to mRNA (Mercer et al., 2008; Quinn and Chang, 2016). However, the functional specificity of lncRNAs remains a subject of debate. Recent studies indicate that lncRNAs can modulate gene transcription in the nucleus, influence mRNA stability and translation, and modify proteins in the cytoplasm. These activities impact various signaling pathways and play crucial roles in numerous cellular and biochemical processes (Yao et al., 2019; Cabili et al., 2011). Additionally, the intrinsic characteristics of cancer cells, such as proliferation capacity, metabolic activity, and apoptosis, are closely linked to lncRNAs (Statello et al., 2021). This evidence suggests that lncRNAs may influence the initiation, metastasis, and colonization of tumor cells at metastatic sites during the metastatic progression of BC. Furthermore, given the pronounced tissue-specific expression of lncRNAs, distinct lncRNA clusters may exert effects at different metastatic sites.

LncRNAs are aberrantly expressed during the progression of BC and exert regulatory functions through diverse mechanisms and factors. This review aims to elucidate the molecular mechanisms and current understanding of lncRNA-mediated multimodal regulation in BC progression (Amelio et al., 2021).

## 2 Generation, function, and classification of lncRNA

### 2.1 LncRNA generation

More than 80% of the human genome is transcribed to produce RNA or engage in chromatin-related activities, while less than 2% of protein-coding genes are transcribed to form mRNA. The majority of these transcripts are lncRNAs (Herman et al., 2022; ENCODE Project Consortium, 2012). LncRNAs are predominantly localized in the nucleus, although they are also present in the cytoplasm. They are transcribed by RNA polymerase II (RNAP II) from intergenic, exonic, and intronic regions of the chromatin, exhibiting lower expression levels compared to protein-coding genes, limited primary sequence conservation, and undergoing splicing, capping, and polyadenylation. Approximately 98% of lncRNAs undergo splicing and are flanked by typical splicing sites (GT/AG), sharing similar splicing signals with protein-coding genes (Derrien et al., 2012). The transcription and processing of lncRNAs are analogous to those of mRNAs, although recent studies have highlighted differences in these processes, which are closely associated with lncRNA expression and functional localization.

### 2.2 LncRNA functions

Through genomic and cellular analysis methods, lncRNAs have been demonstrated to possess multiple functions during the differentiation and proliferation of embryonic stem cells (Guttman et al., 2009). Existing studies have shown that lncRNAs participate in various cellular regulatory processes and play significant roles at different stages. Their influence on subcellular localization even determines functional characteristics (Geisler and Coller, 2013; Chen, 2016). Nuclear lncRNAs interact with chromatin modification complexes, RNA-binding proteins (RBPs), or trans-acting factors to regulate the expression of coding genes. In contrast, cytoplasmic lncRNAs primarily exert post-transcriptional regulatory functions, such as mRNA degradation and protein modification following translation (Chen et al., 2020; Batista and Chang, 2013). LncRNAs serve as molecular vectors to achieve these functions through diverse mechanisms. For instance, Xist has recently been identified as a molecular scaffold that binds to binding proteins to form ribonucleoprotein (RNP) complexes, which are implicated in developing autoimmune diseases (Dou et al., 2024). Acting as a “molecular sponge,” LINC00680 adsorbs miR-423-5p to modulate the expression of PAK6 in esophageal squamous cell carcinoma (ESCC) (Xue et al., 2022). Additionally, lncRNA P53RRA can directly interact with the G3BP1 protein in the cytoplasm, regulating the transcription of metabolic genes, promoting ferroptosis, and inhibiting tumor progression (Mao et al., 2018). With the advancement and maturation of genetic technologies, other mechanisms by which lncRNAs influence cancer progression are gradually being elucidated, and new evidence of lncRNAs’ involvement in human diseases continues to emerge (Ferrer and Dimitrova, 2024; Liu S. J. et al., 2021).

### 2.3 Classification of lncRNAs

**FIGURE 1:**
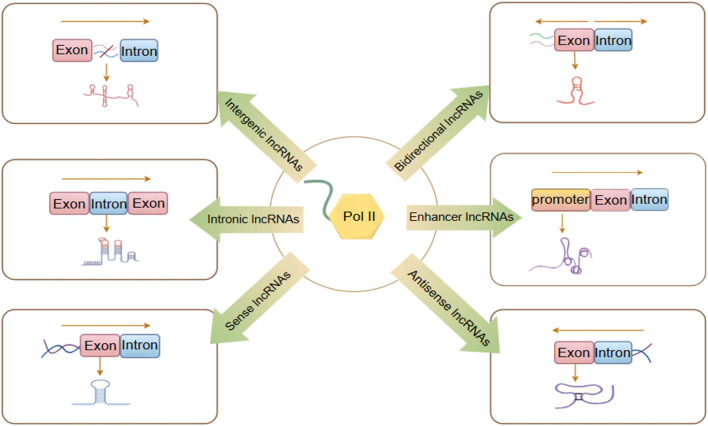
LncRNAs are classified into six different categories. By Figdraw.

Currently, research on lncRNAs remains incomplete. Various studies categorize lncRNA differently, classifying it into distinct categories shown in Figure 1 based on its transcription site transcription direction, and functional characteristics (Tsagakis et al., 2020).

1. Intergenic lncRNAs (lincRNAs) arise from the intergenic regions situated between protein-coding genes, without overlapping with any other genes. This category also encompasses very long intergenic lncRNAs (vlincRNAs), which are transcribed from extensive intergenic regions (ranging from 50 to 700 kilobases), exemplified by Nostrill and vlincRNA VAD (Sharmin et al., 2024; Lazorthes et al., 2015).
2. Intronic lncRNAs(lincRNAs) are generated by transcribing introns within protein-coding regions and typically undergo alternative splicing. This process can facilitate stable transcription and cis-regulation of coding genes, exemplified by ci-ankrd52 and ANRASSF1 (Li X. et al., 2021; Beckedorff et al., 2013).
3. Sense lncRNAs are generated within the exon regions of protein-coding genes. While some protein-coding exons overlap with mRNA and are retained, the majority lack functional open reading frames and are incapable of undergoing protein translation, exemplified by lncRNA GAS5 (Tu et al., 2023).
4. Antisense lncRNAs are transcribed in the reverse orientation relative to protein-coding or non-protein-coding genes, primarily by RNA polymerase III (RNAP III), exemplified by HIFAL and ZNF561-AS1 (Zheng et al., 2021; Si et al., 2021).
5. Bidirectional lncRNAs are transcribed in both directions from the vicinity of the transcription initiation site of protein-coding genes, typically within a distance of less than 1,000 base pairs. These lncRNAs may exhibit biological functions similar to those of their corresponding mRNA partners, as exemplified by LINC00882 (Peralta-Alvarez et al., 2024).
6. Enhancer or promoter lncRNAs are transcribed from enhancer or promoter regions. This category also encompasses transcripts derived from recently identified enhancer clusters, known as Super Enhancer lncRNAs (SE-lncRNAs), exemplified by Carmn and LINC01503 (He et al., 2023; Shen et al., 2020).

## 3 LncRNAs can inhibit or promote BC metastasis

**FIGURE 2:**
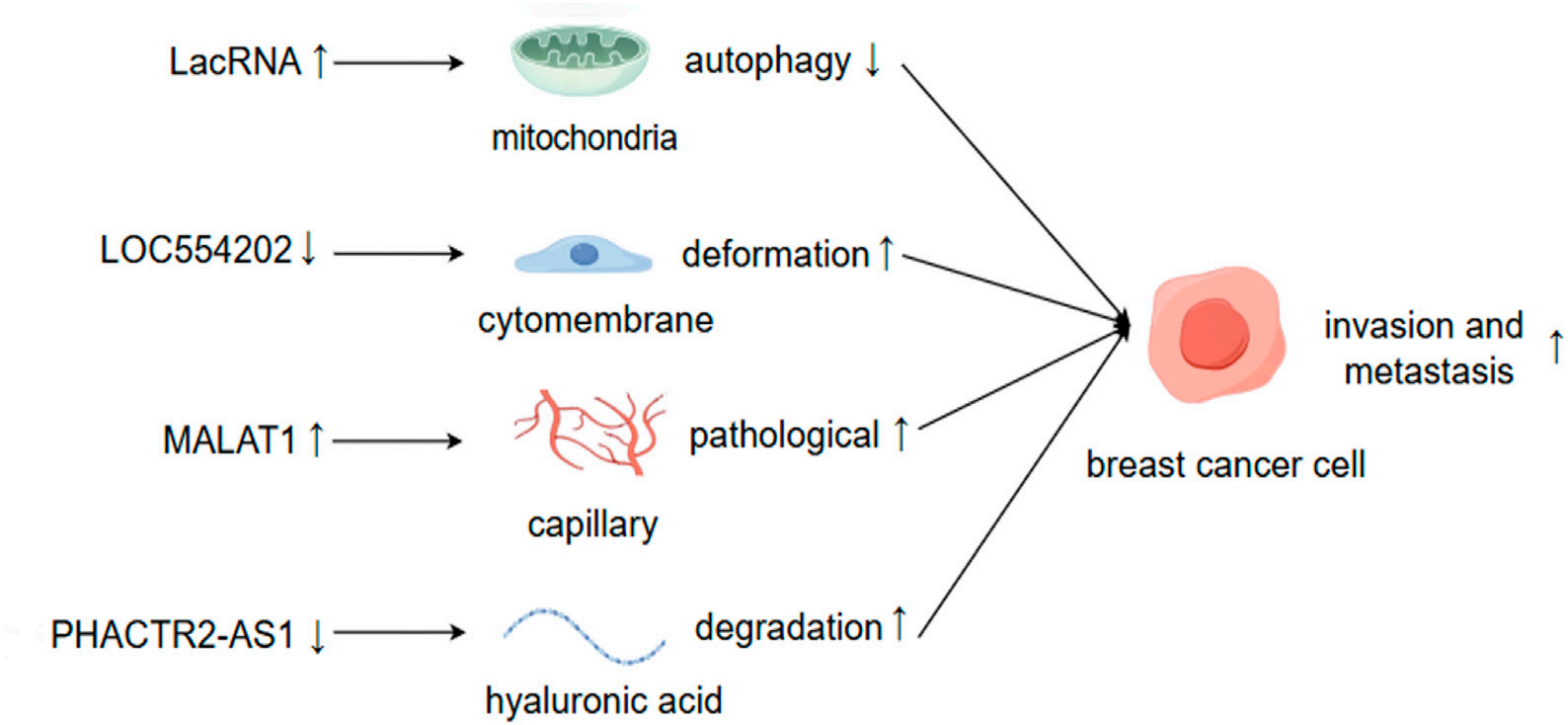
LncRNAs facilitate BC invasion and metastasis through the modulation of both intracellular and extracellular alterations. By Figdraw.

LncRNAs comprise a large family with diverse types and functions (Figure 2). According to the current research, different lncRNAs, or even the same lncRNAs, may exhibit either synergistic or opposing effects in regulating BC metastasis due to variations in their regulatory mechanisms and modes of action (Guzel et al., 2020).

The expression levels of lncRNAs may increase or decrease during tumor metastasis, and the causal relationship between these changes and metastasis remains to be elucidated (Mondal and Meeran, 2020). Generally, lncRNAs that promote cancer metastasis tend to be upregulated in tumors, and silencing these lncRNAs typically suppresses tumor metastasis or induces apoptosis. For instance, lncRNA BRE2 is upregulated in metastatic BC, preventing the interaction between NICD1 and WWP2, thus enhancing the stability of NICD1, activating Notch signaling, and driving BC progression and lung metastasis (Zhang Z. et al., 2023). Conversely, lncRNAs that inhibit cancer metastasis are often downregulated in tumors, and the inactivation of these lncRNAs frequently increases the likelihood of metastasis. Upon cleavage by RNase, LINC00478 undergoes polyadenylation to generate mature cytoplasmic RNA (LacRNA). The 61-140-nucleotide region at the 5′terminus of LacRNA can competitively bind to the PHB domain of PHB2, which interacts with the autophagy recognition protein LC3 on the surface of autophagosomes, thereby inhibiting the autophagic degradation of PHB2 protein in mitochondria. The complex formed by LacRNA and PHB2 directly binds to c-Myc and promotes its degradation, leading to the inhibition of the oncogene Myc and, consequently, the suppression of BC metastasis (Guo et al., 2023).

Existing studies employ various methodologies and technologies, utilizing multiple observation indicators. Consequently, the same lncRNA may exert similar or opposing roles in regulating diverse biological functions, such as cellular drug resistance, the tumor microenvironment, and the cell cycle of BC metastasis. The ultimate inhibitory or promoting effect likely hinges on the predominant factors (Augoff et al., 2012; Sossey-Alaoui et al., 2007). LOC554202 stands out as one of the initial lncRNAs identified for its regulatory role in BC metastasis (Kurisu et al., 2005). Katarzyna Augoff et al. demonstrated that LOC554202, acting as the host gene of miR-31, modulates its transcriptional level. This interaction confers LOC554202 with a significant role in BC metastasis. Promoter methylation associated with LOC554202 reduces the expression of both LOC554202 and miR-31 in triple-negative BC (TNBC) cell lines, thereby influencing the expression and activity of the downstream target molecule WAVE3 during the invasion-metastasis cascade. The expression level of WAVE3 is significantly correlated with BC progression. LOC554202 primarily regulates miR-31, affecting the formation of membrane fold types, thus controlling cell movement—a critical step in BC metastasis. Yongguo Shi et al. reported that the expression of LOC554202 in BC tissues correlates positively with tumor size and clinical stage, and it is overexpressed in two types of triple-negative BC cells (MDA-MB-231 and MDA-MB435S), which contrasts with Katarzyna’s findings (Shi et al., 2014). The same lncRNA can exhibit markedly different functions across various tumor cells and tissues. For instance, lncRNA PHACTR2-AS1 (PAS1) promotes the proliferation and metastasis of hepatocellular carcinoma (HCC) and gastric cancer cells (Chen et al., 2019; Chen et al., 2018). However, the opposite effect was observed in BC organizations. EZH2-mediated methylation results in the loss of PHACTR2-AS1 expression, enhanced ribosome and protein synthesis, and increased genomic instability, ultimately contributing to the development of BC (Chu et al., 2020). PAS1 can also reduce the degradation of hyaluronic acid in the extracellular matrix and inhibit the growth and metastasis of BC by suppressing PH20 (Fu et al., 2022). MALAT1 is a highly expressed long non-coding RNA (lncRNA) in cancer tissue, primarily functioning as an oncogene (Goyal et al., 2021). A study of 20 randomly selected pairs of BC samples found that the expression of MALAT1 in tumor tissues was significantly higher than that in normal non-cancerous tissues. MALAT1 may regulate the activity of endothelial cells through the miR140-5p-JAG1/VEGFA pathway, thereby generating new pathological blood vessels and promoting BC invasion and metastasis (Huang et al., 2018; Liu J. et al., 2023). However, other studies have demonstrated that the downregulation of MALAT1 expression is associated with the proliferation and metastasis of BC, indicating its role as a tumor suppressor. Jongchan Kim et al. employed more rigorous research methods to show that MALAT1 inhibits BC metastasis in various models and can inhibit or even inactivate the transcriptional activity of the transcription factor TEAD (Kim et al., 2018). Gene silencing strategies employing short hairpin RNA (shRNA), small interfering RNA (siRNA), or antisense oligonucleotides (ASOs) without concurrent specific gene verification can complicate the interpretation of phenotypic outcomes following the knockdown of long non-coding RNA (lncRNA) expression. This ambiguity arises because the resultant phenotypes may not be distinctly attributable to the silencing of the targeted lncRNA but rather to the attenuation of other associated genomic elements. For instance, MALAT1 predominantly resides in the nucleus, implying that the application of these various silencing techniques could engender unintended genome-wide effects, particularly concerning nuclear lncRNAs (Abo-Saif et al., 2023). Consequently, the observed phenotypes may stem from the interference with unintended genes rather than the intended targets, thus elucidating the phenomenon wherein identical lncRNAs yield divergent phenotypic consequences (Bassett et al., 2014; Lin et al., 2017). LncRNAs are potential targets for disease treatment and prognosis, and their regulation of BC metastasis is not direct. Clarifying their inhibitory or promoting effects, as well as the interactions between them, is of significant importance for the research and development of lncRNA-based molecular targeted drugs (Yip et al., 2021).

The mechanism by which lncRNAs regulate gene expression remains incompletely understood. A substantial body of research has demonstrated that lncRNAs can interact with various molecules, including DNA, transcription factors, mRNA, miRNA, and proteins (Chen Y. et al., 2021). However, there are relatively few reports on the regulatory relationships among lncRNAs. For instance, Fatemeh Khani Habibabadi et al. discovered a potential feedback loop between HOTAIR and MALAT1. Specifically, HOTAIR downregulates MALAT1 through the sequestration of miR-217. The reduced expression of MALAT1 facilitates its binding to miR-1, thereby promoting HOTAIR expression and forming a positive feedback loop (Khani-Habibabadi et al., 2022). Currently, there is limited research on the interactions between lncRNAs in the context of BC progression. Studies have shown that XIST and MALAT1 exhibit opposing expression patterns in cancer tissues and TNBC cells. In the regulation of BC progression, XIST can counteract the oncogenic effects of MALAT1, establishing a novel immunomodulatory network: the MALAT1/XIST/miR-182-5p/PD-L1 axis (Samir et al., 2021). These findings indicate that lncRNA-lncRNA interactions play a crucial role in cellular functions and gene expression. Identifying these interactions can contribute to a deeper understanding of the underlying mechanisms and the discovery of therapeutic targets (Qian et al., 2019; Singh S. et al., 2022). The structural flexibility of lncRNAs and the limitations of conventional assay techniques have hindered our comprehension of their structure and spatial roles. However, with the ongoing advancements in high-throughput sequencing and other cutting-edge technologies, the study of lncRNA-lncRNA interactions is poised to transform our understanding of their roles in cancer and open new avenues for investigating BC metastasis.

## 4 LncRNAs affect BC metastasis by regulating the cell cycle

**FIGURE 3:**
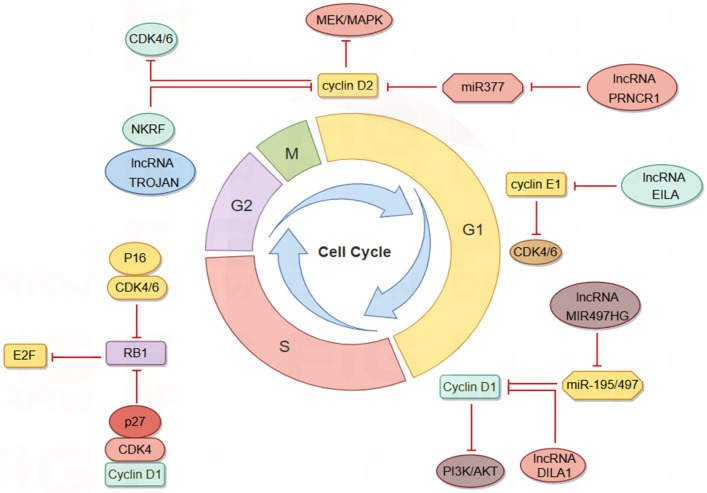
LncRNAs modulate the cell cycle of BC cells via interactions with Cyclin/CDK complexes. By Figdraw.

The cell cycle mechanism is primarily regulated by cyclins and cyclin-dependent kinases (CDKs), which are responsible for driving cell growth and proliferation (Hafner et al., 2019; Malumbres and Barbacid, 2009). This regulatory mechanism is frequently impaired in cancer. CDK4/6 inhibitors (CDK4/6i) have been effectively utilized in the treatment of hormone receptor-positive (HR+)/human epidermal growth factor receptor 2-negative (HER2-) metastatic or advanced BC patients, demonstrating the ability to delay cancer progression and enhance patient quality of life. However, these inhibitors also exhibit cytotoxic side effects and can lead to the development of cellular resistance over time (Hafner et al., 2019; Klein et al., 2018; Lynce et al., 2018; Turner et al., 2019). Research indicates that lncRNAs play a role in regulating cyclins, CDKs, and other cell cycle regulators shown in Figure 3. Consequently, cell cycle-specific lncRNA-targeted therapies are anticipated to emerge as potential alternative treatments in the future (Kitagawa et al., 2013).

LncRNAs influence cyclin expression by acting as competing endogenous RNAs (ceRNAs). Cyclin D1 is overexpressed in approximately 50% of BC cells and is associated with the risk of BC progression and metastasis (Fu et al., 2004; Yu et al., 2008). Cyclin D1 regulates various biological functions, including cell proliferation and migration, angiogenesis, maintenance of stem cell activity, and regulation of microRNA (miRNA) subsets, and serves as a key regulator of the G1-S transition (Yu et al., 2013). MIR497HG was initially identified as dysregulated in bladder cancer, and subsequent studies have shown that it primarily functions as a tumor suppressor in tumors (Zhuang et al., 2020; Eissa et al., 2019). Deletion of MIR497HG downregulates the expression of miR-195/497, increases cyclin D1 levels, and inhibits the PI3K-AKT signaling pathway. Downregulation of MIR497HG mediates the involvement of cyclin in the mechanism of tamoxifen resistance, thereby facilitating BC progression and metastasis (Tian et al., 2023). PRNCR1, another highly expressed lncRNA in BC tissues, may inhibit cell cycle progression by regulating the phosphorylation levels of CHK2 and AKT (Pang et al., 2019). Knockdown of PRNCR1 downregulates the expression of cyclin D2, induces G0/G1 phase arrest of the BC cell cycle, and inhibits BC progression through the miR-377/CCND2/MEK/MAPK axis (Ouyang et al., 2021).

LncRNA can directly interact with cyclin to influence its stability and activity. Cyclin E1, encoded by the CCNE1 gene, binds and activates CDK2, leading to the deactivation of Rb through phosphorylation and promoting the transition from the G1 phase to the S phase of the cell cycle (Chu et al., 2021; Hwang and Clurman, 2005; Zeng et al., 2023). Overexpression of cyclin E1 in BC is associated with poor prognosis and drug resistance (Caldon et al., 2012; Scaltriti et al., 2011). LncRNA EILA stabilizes cyclin E1 by directly affecting the phosphorylation site at the C-terminal of cyclin E1, preventing the binding of cyclin E1 to the ubiquitin ligase FBXW7, thus inhibiting ubiquitination-mediated proteasomal degradation. This stabilizing effect results in increased cyclin E1 expression and contributes to the development of BC resistance to CDK4/6 inhibitors (Cai et al., 2023). DILA1, a nucleolar lncRNA similar to MALAT1, contains a hairpin A structure that binds directly to the Thr286 phosphorylation site of cyclin D1, leading to a decrease in phosphorylated cyclin D1 (Thr286) and subsequent reduction in nucleoplasmic translocation and degradation of cyclin D1. By upregulating cyclin D1, DILA1 accelerates G1 phase progression, which is essential for inducing cell proliferation and tamoxifen resistance (Shi et al., 2020).

CDKs are essential regulators of the cell cycle, playing a pivotal role in cellular processes. Their activation depends on the binding of cyclins, which enables their kinase activity and includes both cell cycle-associated CDKs and those linked to transcription (Chou et al., 2020). These influential kinases integrate vital signal transduction pathways that promote tumor growth, positioning them as promising targets for novel molecular therapies. Furthermore, CDKs can interact with lncRNAs during tumor progression, underscoring their multifaceted involvement in cancer biology (Siddique et al., 2024). Given the structural and functional similarities between CDK4 and CDK6, which facilitate functional compensation, studying these enzymes together is crucial for enhancing our understanding of cancer treatment strategies (Pavletich, 1999). The INK family and the CIP/KIP family serve as kinase inhibitors controlling cell cycle transitions, primarily through the inhibition of CDK4/6 to modulate the cell cycle (Bury et al., 2021). P16, a ubiquitously expressed member of the INK family, forms complexes with CDK4 and CDK6, thereby reducing the formation of the CDK4/6-cyclin D complex and inhibiting the phosphorylation of retinoblastoma protein 1 (RB1). Reduced RB1 phosphorylation enhances its affinity for the E2F transcription factor family, increasing the inhibitory effect on E2F upon binding and suppressing transcription (Hiebert et al., 1992; Sellers et al., 1995). While CIP/KIP proteins are generally regarded as CDK inhibitors, research by Seth M. Rubin et al. indicates that p27, a member of the CIP/KIP family, functions as an allosteric activator. Tyrosine-phosphorylated p27 activates CDK4 and binds to the CDK4-cyclin D1 complex, inducing alterations in the ATP binding site and releasing kinase-activating fragments, leading to the inhibition of the cell cycle by phosphorylated RB (Guiley et al., 2019). To address resistance to CDK4/6 inhibitors, efforts are underway to identify various biomarkers to predict CDK4/6 sensitivity and to develop novel combination drug strategies (Pandey et al., 2019; Rugo et al., 2021). Given the diverse mechanisms by which lncRNAs regulate the cell cycle, the relationship between lncRNAs and CDK4/6 inhibitors appears very promising (Guiducci and Stojic, 2021). Research has shown that the expression of lncRNA TROJAN is significantly elevated in estrogen receptor-positive BC (ER^+^BC) cells, correlating with reduced patient survival rates. Both *in vivo* and *in vitro* studies have demonstrated that the combination of Anti-TROJAN ASO with CDK4/6 inhibitors more effectively inhibits tumor growth compared to the use of either inhibitor alone, indicating a significant synergistic effect. Mechanistic investigations have revealed that TROJAN binds to NKRF (NF-κB inhibitor), indirectly activating transcription factors in the NF-κB signaling pathway, which is implicated in BC cell proliferation, metastasis, and endocrine therapy resistance (Devanaboyina et al., 2022). In the presence of CDK4/6 inhibitors, the TROJAN-NKRF complex upregulates the expression of CDK2, enabling BC cells to enter the S phase via an atypical pathway and continue the cell proliferation process. The regulatory mechanism of TROJAN-NKRF-CDK2 offers a plausible explanation for CDK4/6 inhibitor resistance and identifies a potential target for overcoming this resistance (Jin et al., 2020; Jin et al., 2019). Identifying cancers that depend on a single CDK for proliferation, enhancing the diversity and interchangeability of selective inhibitors are crucial for developing safe and effective drugs (Morrison et al., 2024). CDK7 functions as a CDK-activated kinase (CAK) responsible for activating the kinase activities of CDK1, CDK2, CDK4, and CDK6, thereby regulating downstream cell cycle processes. Additionally, CDK7 collaborates with cyclin H and MAT1 to form the core of transcription factor IIH (TFIIH), which activates the C-terminal domain (CTD) of RNA polymerase II, facilitating transcription (Sava et al., 2020; Li et al., 2022). Given its pivotal roles in cell cycle regulation and transcription, CDK7 represents a promising target for cancer drug development. Furthermore, CDK7 mediates the activity of the ER α through phosphorylation at serine 118, and elevated blood estrogen levels are associated with an increased risk of BC. This suggests that CDK7 inhibitors (CDK7i) hold significant potential in BC therapy (Song et al., 2024; Chen et al., 2002). LIMD1-AS1 is a lncRNA associated with super-enhancers (SEs) and acts as an oncogene specifically overexpressed in glioma. It promotes the proliferation and invasiveness of glioma cells and exhibits a negative correlation with patient survival. CDK7 enhances the transcription of LIMD1-AS1 by phosphorylating the transcription coactivator MED1, which subsequently binds to HSPA5 and activates the interferon signaling pathway (Chen et al., 2023). Other studies have utilized esophageal squamous cell carcinoma (ESCC) as a model to successfully predict several SE-associated competing ce-lncRNAs using a computational method named GloceRNA. Additionally, these studies have demonstrated that the specific CDK7 inhibitor THZ1 downregulates SE-associated ce-lncRNAs by inhibiting transcription factors, such as reducing the expression of LINC00094 in ESCC cells (Wang et al., 2020). While there is substantial evidence supporting a reciprocal regulatory relationship between lncRNAs and CDK7 in various tumor types, a significant gap persists: no direct evidence has yet been established linking lncRNAs associated with BC metastasis to the regulation of CDK7.

Various lncRNAs influence the cell cycle by modulating the activity of CDKs through diverse mechanisms, and these regulatory effects may play a crucial role in the progression and metastasis of BC. The impact of lncRNAs on cyclin kinase activity is a key aspect of the complex pathogenesis of cancer, and further experimental data are required to elucidate the specific mechanisms of this interaction.

## 5 Significance of lncRNAs in tumor microenvironment

**FIGURE 4:**
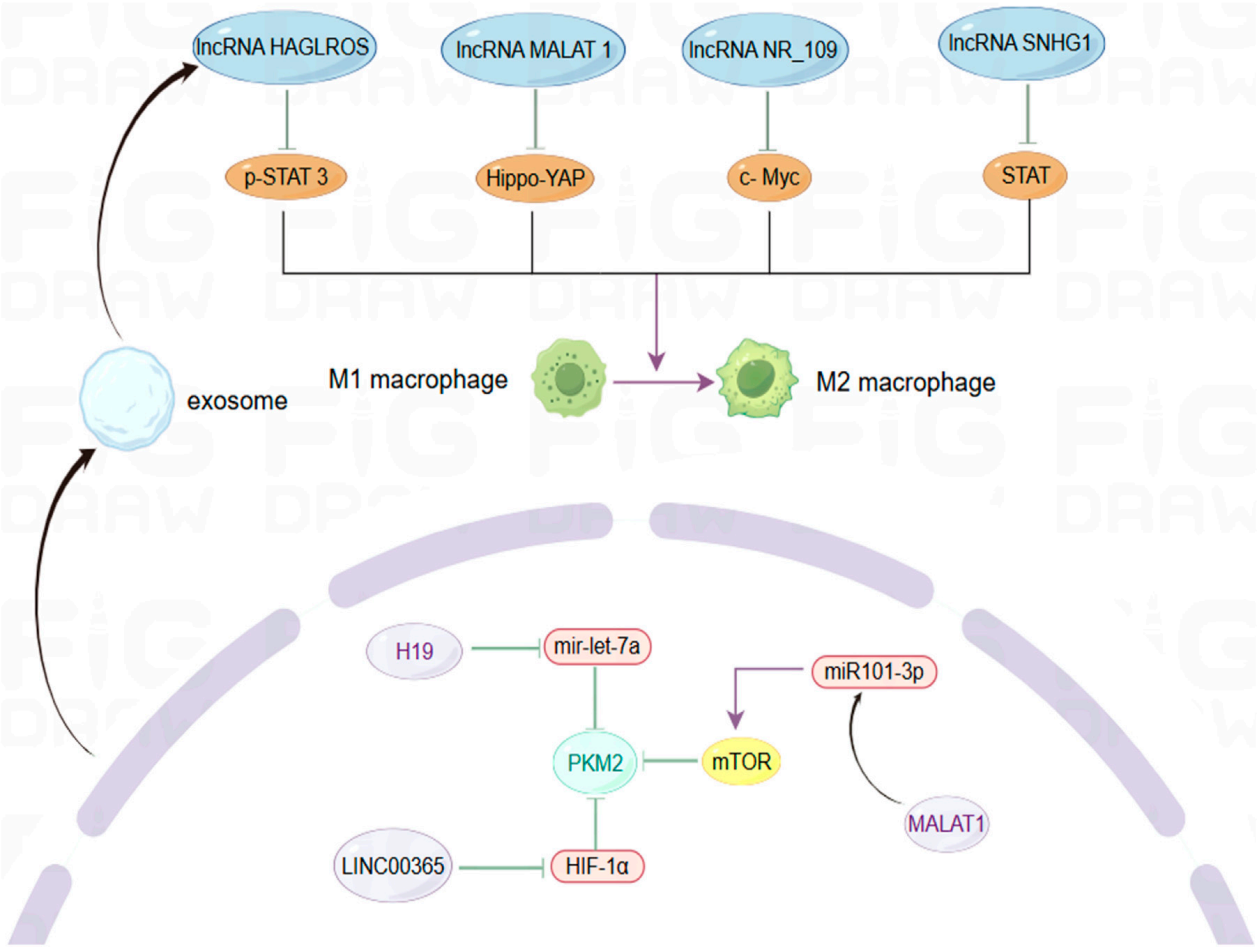
The role of lncRNAs in modulating macrophage phenotype and metabolic enzyme expression within the BC microenvironment. By Figdraw.

The tumor microenvironment (TME) encompasses a sophisticated network comprising cancer cells, non-cancerous cells (including stromal and immune cells), and the molecules secreted both before and following tumor development. This intricate system significantly influences tumor initiation, invasion, metastasis, inflammatory responses, proliferation maintenance, and immunosuppression (Xiao and Yu, 2021). As tumors expand, they release active factors and generate metabolites that modify the TME’s composition (Figure 4), thereby further facilitating tumor progression (Economopoulou et al., 2020). The metastatic potential and prognosis of BC are intimately linked to alterations in the TME, underscoring the critical need to elucidate the compositional changes and regulatory mechanisms within this environment (Qian et al., 2011).

As key players among immune cells in the TME, tumor-associated macrophages (TAMs) exhibit dynamic changes in their polarization states, which can be influenced by various factors (Murray et al., 2014). Research has primarily identified two phenotypes of macrophages: M1 and M2. M1 macrophages display pro-inflammatory and anti-tumor activities, while M2 macrophages exhibit anti-inflammatory and pro-tumor behaviors. Despite the limitations of this classification, it remains valuable for understanding tumor immunity (Van den Bossche et al., 2017). Monocytes recruited from the peripheral circulation of the TME predominantly differentiate into M2 macrophages, which secrete angiogenic factors, cell proliferation factors, immunosuppressive factors, and stromal proteolytic enzymes, thereby supporting cancer cell growth and metastasis (Hagemann et al., 2005; Leek et al., 1996). Consistent with this, lncRNAs have been identified as regulators of TAMs polarization across various cancer types, often contributing to pro-tumor activities, often exerting pro-tumor effects. For instance, exosomes secreted by TAMs can transfer lncMMPA, leading to the formation of M2-type macrophages. The binding of highly expressed lncMMPA to miR-548s in hepatocellular carcinoma enhances aerobic glycolytic pathways and cell proliferation (Xu et al., 2022). Similarly, lncRNA-PACERR is overexpressed in TAMs derived from pancreatic ductal adenocarcinoma (PDAC) and binds to miR-671-3p *in vitro*, releasing KLF12 and activating the AKT/c-myc pathway, thus promoting M2 macrophage polarization (Liu et al., 2022). BC has a notably higher proportion of TAMs within the tumor, sometimes accounting for up to 50% of the tumor volume (Wagner et al., 2019). Although the number of TAMs varies significantly among individuals, they consistently contribute to BC progression, highlighting the critical role of macrophage phenotype in BC invasion and metastasis (Biswas et al., 2013; Xiao et al., 2022). LncRNA SNHG1 regulates macrophage phenotype via the STAT pathway, promoting BC metastasis and angiogenesis at the metastatic site (Zong et al., 2021). LncRNA NR_109 has emerged as a critical factor in macrophage polarization, competing with JTV-1 to bind to the C-terminal of FUBP1. This interaction inhibits the ubiquitin-mediated degradation of FUBP1, resulting in a significant increase in c-Myc expression and promoting a shift towards M2-like macrophage polarization (Zhang C. et al., 2023). Emerging evidence suggests that the transformation of TAMs from a pro-tumor to an anti-tumor phenotype could alter the TME and provide a platform for inhibiting tumor growth, proliferation, and metastasis, with lncRNAs being a potential breakthrough direction. The utilization of macrophage phagocytosis to enhance innate immunity has garnered increasing attention in cancer therapy (Chen S. et al., 2021). Recent studies have demonstrated that, in addition to regulating TAM polarization, lncRNAs can also inhibit macrophage phagocytosis and promote cancer cell immune escape. For instance, LINC00460 promotes the overexpression of CD47, which inhibits the recognition and phagocytosis of colorectal cancer (CRC) cells by macrophages, hindering cancer cell clearance (Luo et al., 2024). Similarly, in epithelial ovarian cancer (OC), lncRNA IL21-AS1 mediates the regulation of a novel antiphagocytic protein, CD24, through HIF-1α and/or miR-561-5p, rather than CD47 (Liu et al., 2024). In TNBC, a novel engineered nanoparticle (P-ACD24/CEL + P/shMFN1) synergistically elicits an anti-tumor immune response by blocking CD24, inducing tumor cell apoptosis, and reversing the M2-TAMs phenotype, thereby achieving the effect of combined immunotherapy (Zhao et al., 2023). These findings underscore the significance of enhancing macrophage phagocytosis in immunotherapy, where anti-phagocytosis proteins serve as critical regulators. CD24 and CD47 are frequently overexpressed in solid tumor samples from BC patients, with their expression levels varying across different BC subtypes. Specifically, CD24 expression in fibroadenomas is higher than in phyllodes tumors, while CD47 is predominantly expressed in drug-resistant HER2^+^ BC cells (Ahmed et al., 2022; Zhang B. et al., 2024). Moreover, CD24 is implicated in the invasive and metastatic behavior of cancer cells, with its expression significantly elevated at distant metastatic sites compared to primary tumors (Shipitsin et al., 2007). Additionally, CD24 deletion in mouse models of BC xenotransplantation has been shown to inhibit primary tumor growth and, more importantly, may hinder the colonization and growth of cancer cells in distant organs such as the lungs by reducing the formation of microvessels and lymphatics (Chan et al., 2019). Despite this, targeted therapy against CD24 has yielded promising results. LncRNAs plays a crucial role in regulating the expression of these immune checkpoints through mechanisms such as acting as a “molecular sponge.”

Pyruvate kinase M2 (PKM2) is a critical rate-limiting enzyme in glycolysis that plays a pivotal role in regulating cellular metabolism within the TME. Metabolic reprogramming is a hallmark of cancer cells, and PKM2 is essential for the metabolic phenotype associated with aerobic glycolysis (Hanahan and Weinberg, 2011; Christofk et al., 2008). In normal cells, the dimeric form of PKM2 exhibits low catalytic efficiency and is subject to intricate regulation as a protein kinase (Gui et al., 2013). Unlike mechanisms that manipulate genetic information, this lncRNA influences cancer cell proliferation by modulating the activity of metabolic enzymes, thereby enhancing our understanding of lncRNA function. The altered expression of PKM2 is a significant factor in various cancers, with emerging evidence underscoring the critical role of lncRNAs in regulating this enzyme (Zhu et al., 2021). For instance, lncRNA 495810 is essential in CRC, as it stabilizes PKM2 activity by preventing its degradation through ubiquitination (Cui et al., 2023). Furthermore, the transcription factor YY1 enhances the expression of lncRNA-ARAP1-AS2 and ARAP1, resulting in persistent activation of EGFR, PKM2 transformation, and the accumulation of HIF-1α. This interplay has significant implications, driving the abnormal glycolysis seen in diabetic nephropathy (DKD) and contributing to renal tissue fibrosis (Li et al., 2023). Conversely, lncRNA PWRN1 inhibits hepatocellular carcinoma (HCC) growth, invasion, and metastasis by forming a stable tetramer and increasing PKM2 activity, suppressing aerobic glycolysis and reducing lactate levels (Fei et al., 2024). Additionally, PKM2 is a key mediator in the polarization of macrophages through metabolic reprogramming pathways (Palsson-McDermott et al., 2015). Recent studies have demonstrated that annexin A5 (Anx A5) targets PKM2 to reduce its nuclear translocation, inhibit glycolysis, and shift metabolism towards tricarboxylic acid (TCA) cycle-dependent oxidative phosphorylation (OXPHOS), thus facilitating the transition of liver macrophages from the M1 to the M2 phenotype (Xu et al., 2020). PKM2 fulfills multiple roles in cancer, serving not only as a catalytic enzyme in metabolism, catalyzing the final step of glycolysis, but also as a protein kinase and transcriptional regulator with oncogenic functions, thereby promoting gene transcription and tumor progression (Yang et al., 2011; Yang et al., 2014). By skillfully balancing both metabolic and non-metabolic roles, PKM2 effectively orchestrates the complex process of tumorigenesis. However, the intricacies of its dual role continue to prompt debate, underscoring its relevance in cancer research and potential therapeutic strategies. For instance, PKM2-deficient xenograft tumor models established *in vivo* did not exhibit restricted tumor growth, and similarly, PKM2 knockdown had no significant inhibitory effect on TNBC (Cortés-Cros et al., 2013; Israelsen et al., 2013). Research has demonstrated that the absence of PKM2 triggers a shift in the TNBC metabolic pathway from glycolysis to fatty acid beta-oxidation (FAO), which continues to supply energy for cancer cell activity (Zhang Y. et al., 2024). PKM2 is associated with advanced metastasis, tumor classification, and poor prognosis in BC. Fluctuations in estrogen and progesterone levels before and after menopause influence the expression of glycolysis-related genes, indicating a potential interplay between hormonal changes and PKM2 (Xiao et al., 2020; Ishfaq et al., 2022). MALAT1 regulates BC processes via the miR101-3p/mTOR/PKM2 pathway (Shao et al., 2021). LINC00365 may suppress the expression of key glycolytic enzymes by targeting HIF-1α (Liu B. et al., 2023). These studies have elucidated a novel mechanism of BC-associated lncRNA-mediated regulation of PKM2 activity. Before metastasis, BC secretes small molecules that act on normal cells at the metastatic site, reducing their energy consumption and facilitating metastasis by “sequestering” cancer cells in pre-metastatic niches (Fong et al., 2015). Following BC metastasis, cancer cells at the metastatic site exhibit altered metabolic processes to adapt to the distinct TME. BC cells with liver metastasis display aerobic glycolysis-dependent metabolic characteristics, whereas BC cells from bone or lung metastasis tend to rely on OXPHOS (Dupuy et al., 2015; Biondini et al., 2024). Some studies have also demonstrated that the upregulation of OXPHOS during BC invasion and migration is a transient phase, and aerobic glycolysis remains predominant after colonization. Energy metabolism is a critical and dynamic component throughout BC metastasis process (Liu YM. et al., 2023). It is essential to deepen our understanding of the mechanisms of energy consumption at various stages of progression and across different metastatic sites. Although PKM2 is a promising therapeutic target, its effectiveness as a standalone agent in targeted small molecule therapy is somewhat limited. The strategic combination of PKM2 inhibitors with other anticancer metabolic drugs holds great promise for effectively disrupting metabolic processes and, as a result, halting tumor growth. Additionally, the unique characteristics of lncRNAs and their regulatory influence on metabolic pathways provide exciting new avenues for targeted molecular therapies in BC.

## 6 LncRNAs modulate the drug resistance of BC cells through the regulation of autophagy

Autophagy is a highly conserved self-protective mechanism present in eukaryotic cells, playing an essential role in maintaining cellular health. It functions by degrading and recycling damaged organelles and residual proteins within the cytoplasm, thereby mitigating their detrimental effects on cellular function (Debnath et al., 2023). Remarkably, even in the presence of cancer, the integrity of the autophagy machinery remains largely intact at the DNA level, with a significantly lower frequency of mutations observed in autophagy-related protein (ATG) genes compared to normal tissues (Levine and Kroemer, 2019). It suggests that mutations in ATG genes are not a primary driver of disease progression. Nevertheless, aberrant autophagy has garnered significant attention in oncology, indicating that certain regulatory factors or pathway interaction elements may modulate the autophagy pathway, leading to either tumor suppression or tumor promotion.

Extensive research has shed light on the complex and often ambiguous role of autophagy in BC, demonstrating that its effects are influenced by key factors such as tumor stage, oncogenic genes, and changes in the microenvironment. Beclin 1, an autophagy-related gene that exerts a negative regulatory effect on BC, was the first to establish a specific connection between autophagy and BC (Liang et al., 1999). In the early stages of cancer, appropriate levels of autophagy can facilitate DNA repair and regulate cell division, thereby counteracting the stimulatory effects of oncogenes and preventing tumor progression. However, as the tumor advances to later stages, the products of autophagic degradation are exploited by cancer cells to support their energy metabolism and biosynthetic requirements, which promotes rapid tumor growth and proliferation, thus contributing to the pro-tumorigenic role of autophagy in cancer progression (Rybstein et al., 2018; Wang et al., 2023). Numerous studies have confirmed the involvement of lncRNAs in the development of drug resistance in BC, and their interaction with autophagy plays a crucial role in enhancing multidrug resistance and advanced metastasis. The expression levels of lncRNAs are closely associated with the emergence of clinical drug resistance in various treatment modalities, including endocrine therapy, chemotherapy, and targeted therapy. This concerning trend reveals the complex interplay of multiple signaling pathways, highlighting the urgent need to address lncRNA-driven mechanisms in the battle against drug-resistant BC. For instance, lncRNAs may enhance tumor resistance via autophagy mediated by the ERK/mTOR, AKT/mTOR, or TGF-β/Smad3 pathways (Li et al., 2019; Xing et al., 2024; Liu X. et al., 2023). Tamoxifen (TAM) is an efficacious medication for the treatment of BC across all clinical stages; however, the development of long-term drug resistance has constrained its therapeutic efficacy. The transcription factor EB (TFEB), lactate dehydrogenase A, and certain lysosome-associated protective proteins can induce endocrine resistance via the autophagy of BC cells. Conversely, vitamin D may potentiate the sensitivity of drug-resistant BC cells to TAM by inhibiting autophagy (Boretto et al., 2023; Das et al., 2019; Actis et al., 2021; Li Y. et al., 2021). LncRNA H19 has been identified as an oncogene in BC, exhibiting a strong correlation with ER expression. Additionally, H19 plays a role in the mechanisms of autophagy or drug resistance in various malignancies, including liver, colorectal, and BC. The H19/SAHH/DNMT3B axis facilitates TAM resistance in BC by modulating DNA methylation and activating autophagy (Basak et al., 2015; Wang et al., 2019). Paclitaxel (PTX) is a frontline chemotherapeutic agent for BC, primarily targeting mitotic processes. Nonetheless, the emergence of drug resistance is inevitable following prolonged administration. In the context of BC chemotherapy resistance, autophagy is a survival strategy for cancer cells adapting to extended treatment regimens. Therefore, inhibiting autophagy can render chemoresistant cancer cells resensitized to chemotherapy agents (Abu et al., 2019). LncRNAs, acting as a regulator of autophagy, have emerged as a promising therapeutic target for overcoming chemotherapy resistance. Indeed, existing research has shown that targeting lncRNAs can inhibit autophagy-mediated activation, thereby enhancing the efficacy of chemotherapy in BC cells. Specifically, knocking down lncRNA DDIT4-AS1 alleviates the suppression of the DDIT4-mTOR signaling pathway, significantly inhibits autophagy, and restores the sensitivity of TNBC to paclitaxel (Jiang et al., 2023). Additionally, the downregulation of lncRNA OTUD6B-AS1, functioning as a competing ceRNA, modulates the expression of miR-26a-5p, inhibits autophagy, and improves paclitaxel-induced cytotoxicity (Li P. P. et al., 2021). Conversely, lncRNA EGOT, a clinical biomarker for assessing paclitaxel sensitivity, enhances paclitaxel sensitivity by upregulating ITPR1 expression and promoting autophagy (Xu et al., 2019). Trastuzumab represents the initial first-line targeted therapy for HER2-overexpressed metastatic BC; however, over 50% of patients receiving this treatment eventually develop resistance. Resistance to HER2-targeted therapies can arise through various mechanisms, including HER2 heterogeneity, tumor immune responses, activation of compensatory pathways, HER2 site mutations, or epitope deletions. Clinical studies have demonstrated that HER2 positivity in primary tumors is associated with a significantly increased risk of central nervous system (CNS) metastasis. Given the limited ability of trastuzumab to penetrate the blood-brain barrier, the CNS may serve as a potential sanctuary for cancer cells, potentially exacerbating drug resistance (Lin and Winer, 2007; Swain et al., 2023). Research has shown that lncRNA ZNF649-AS1 enhances ATG5 transcription by binding to PTBP1, promoting autophagy and contributing to trastuzumab resistance (Han et al., 2020). This novel perspective on the lncRNA-autophagy-cancer axis highlights a promising direction for identifying potential drug resistance mechanisms and enhancing the efficacy of cancer treatments, paving the way for more targeted therapeutic approaches.

## 7 Conclusion and future perspectives

This review comprehensively explores the characteristics and mechanisms by which lncRNAs facilitate the invasion and metastasis of BC. It provides an in-depth analysis of the regulatory interactions between lncRNAs and various factors, including cell cycle mechanisms, TME, and autophagy-related drug resistance shown in Table 1. Notably, the mechanisms by which certain lncRNAs mediate BC metastasis are not completely independent. For instance, lncRNAs not only exert influence over tumor cells through cyclins but also play a significant role in modulating the TME (Deng et al., 2018; Goel et al., 2017). Similarly, lncRNAs regulate BC drug resistance through multiple pathways, with their molecular basis being a complex regulatory network influenced by various processes and factors. Investigating the molecular foundation and mechanisms underlying these interactions will not only enhance our understanding of lncRNA-mediated BC metastasis but also highlight the potential of specific lncRNAs as prognostic biomarkers for BC progression and treatment (Miao et al., 2021).

**TABLE 1:** Summary of recent updates regarding the actions and regulatory targets of various lncRNAs in BC progression.

Pathway of action for lncRNAs -on	LncRNA	Dysregulation	Target	References
Cell cycle	MIR497HG	↓	↓ miR-195/497,	Tian et al. (2023)
			↓ PI3K-AKT	
	PRNCR1	↑	↓ miR-377.	Ouyang et al. (2021)
			↑ MEK/MAPK pathway	
	EILA	↑	↑ cyclin E1,	Cai et al. (2023)
			↓ CDK4/6i	
	DILA1	↑	↑ cyclin D1	Shi et al. (2020)
	TROJAN	↑	↓ NKRF,	Devanaboyina et al. (2022)
			↑ NF-κB pathway	
	SLC16A1-AS1	↓	↑ miR-182,	Jiang et al. (2022)
			↑ PDCD4	
	SNHG5	↑	↓ miR-299,	Huang et al. (2022)
			↑ BACH1	
	LINC02613	↓	↑ Wnt pathway	Cui et al. (2022)
TME	SNHG1	↑	↑ STAT pathway	Zong et al. (2021)
	NR_109	↑	↑ c-Myc	Zhang et al. (2023b)
	HAGLROS	↑	↓ miR-135b-3p,	Meng et al. (2024)
			↑ p-STAT3	
	MALAT1	↑	↑ Hippo/YAP pathway	Wang et al. (2024)
	PCAT6	↑	↑ VEGFR/AKT/mTOR pathway	Dong et al. (2020)
	IRENA	↑	↑ NF-κB	Liu et al. (2021b)
	Linc00514	↑	↑ Notch pathway	Tao et al. (2020)
	Xist	↑	↓ miR-101,	Zhao et al. (2021)
			↑ C/EBPα and KLF6	
	lincRNA-p21	↑	↓ NF-κB pathway,	Zhou et al. (2020)
			↓ STAT3 pathway	
Autophagy	H19	↑	↑ SAHH,	Wang et al. (2019)
			↑ DNMT3B	
	DDIT4-AS1	↑	↓ mTOR pathway	Jiang et al. (2023)
	EGOT	↓	↓ ITPR1	Xu et al. (2019)
	ZNF649-AS1	↑	↑ PTBP1,	Han et al. (2020)
			↑ ATG5	

As the understanding of lncRNAs in the regulation of BC progression continues to expand, our objective is to systematically synthesize the relevant mechanisms and characteristics underlying these regulatory processes. This synthesis aims to provide a foundational framework for the identification of novel anti-cancer targets and the development of innovative therapeutic strategies for advanced BC within the paradigm of precision medicine. A substantial body of preclinical and clinical research has persistently underscored the potential biological significance and therapeutic value of lncRNAs in the context of cancer treatment. Significant advancements have been achieved in the exploration of lncRNAs within the domain of human genetics; however, numerous scientific gaps and technical challenges persist. The redundancy and functional complexity associated with lncRNAs hinder the establishment of a unified standard for their classification and nomenclature. For instance, a single lncRNA may be designated by different names across various databases and literature (e.g., GAS5 is also referred to as SNHG2), thereby exacerbating the complexity of research outcomes and complicating data integration efforts. Furthermore, the functions of over 90% of lncRNAs remain poorly defined, and the identification of functional lncRNA transcription sites is impeded by several obstacles, including sequence conservation, expression heterogeneity, and technical constraints. Consequently, elucidating the functions of lncRNAs and systematically annotating their transcription sites is crucial for enhancing the understanding of gene regulation. Previous research has indicated that lncRNAs are differentially expressed in the context of cancer development, highlighting their regulatory functions. Nonetheless, their relevance to the occurrence of human diseases has been largely overlooked in clinical genomic analyses. A seminal study by Ganesh et al. has demonstrated for the first time that the deletion of lncRNA CHASERR gene can result in neurodevelopmental disorders. This finding underscores the association of lncRNAs with a broader spectrum of human diseases and raises important questions regarding whether the loss, mutation, or amplification of lncRNAs may contribute to the pathogenesis of cancer, necessitating further investigation (Allou et al., 2021; Ganesh et al., 2024). While certain lncRNAs, such as H19, exhibit potential utility in the diagnosis of BC, it is important to note that their sensitivity and specificity remain inferior to those of traditional protein biomarkers. Additionally, the stability of lncRNAs in the bloodstream is adversely affected by the activity of RNA-degrading enzymes, which poses a challenge to their clinical application (Badowski et al., 2022).

Regarding the therapeutic application of lncRNAs associated with BC metastasis, despite the relative maturity of small siRNAs and antisense oligonucleotides, challenges remain in terms of delivery, stability, and immunogenicity. Excitingly, advancements in RNA therapy technology have enabled researchers to design and synthesize high-affinity artificial lncRNAs (alncRNAs) by utilizing RNA aptamers and lncRNA HOTAIR to specifically target and degrade key oncogenic proteins. While several challenges persist, the emergence of lncRNA-targeted therapeutic strategies signifies a noteworthy advancement, offering potential pathways for more effective interventions in BC metastasis (Cao et al., 2024). Simultaneously, nanotechnology-based drug delivery systems have demonstrated advantages in combination therapy for the treatment of BC. Notably, nanoparticles conjugated with siRNA and chemotherapeutic agents exhibit substantial antitumor effects in both *in vivo*, *in vitro*, and organoid studies (Jiang et al., 2023). Furthermore, the field of small molecule therapy, which targets lncRNAs, has emerged as a promising area of research. This domain seeks to disrupt the interactions between lncRNAs and small molecules, thereby modulating the functional roles of lncRNAs in oncogenesis and therapeutic resistance (Alkan et al., 2024). Considering the tissue and organ-specific nature of lncRNAs, they hold significant potential as diagnostic and prognostic biomarkers, as well as therapeutic targets for evaluating clinical outcomes and guiding targeted therapies. Advances in sequencing, probing, and immunoprecipitation techniques have facilitated a more comprehensive understanding of lncRNAs, and their role in the progression and metastasis of BC is poised to yield imminent breakthroughs.

## References

[B1] Abo-Saif M. A. Ragab A. E. Ibrahim A. O. Abdelzaher O. F. Mehanyd A. B. M. Saber-Ayad M. (2023). Pomegranate peel extract protects against the development of diabetic cardiomyopathy in rats by inhibiting pyroptosis and downregulating LncRNA-MALAT1. Front. Pharmacol. 14, 1166653. 10.3389/fphar.2023.1166653 37056985 PMC10086142

[B2] Abu S. T. M. Samec M. Liskova A. Kubatka P. Büsselberg D. (2019). Paclitaxel’s mechanistic and clinical effects on breast cancer. Biomolecules 9 (12), 789. 10.3390/biom9120789 31783552 PMC6995578

[B3] Actis C. Muzio G. Autelli R. (2021). Autophagy triggers tamoxifen resistance in human breast cancer cells by preventing drug-induced lysosomal damage. Cancers 13 (6), 1252. 10.3390/cancers13061252 33809171 PMC7999102

[B4] Ahmed S. S. Lim J. C. T. Thike A. A. Iqbal J. Tan P. H. (2022). Epithelial-mesenchymal transition and cancer stem cell interactions in breast phyllodes tumours: immunohistochemical evaluation of EZH2, EZR, HMGA2, CD24 and CD44 in correlation with outcome analysis. J. Clin. pathology 75 (5), 316–323. 10.1136/jclinpath-2020-207068 33627375

[B5] Alkan A. H. Ensoy M. Cansaran-Duman D. (2024). Strategic and innovative roles of lncRNAs regulated by naturally-derived small molecules in cancer therapy. Curr. Med. Chem. 31 (40), 6672–6691. 10.2174/0109298673264372230919102758 37921177

[B6] Allou L. Balzano S. Magg A. Quinodoz M. Royer-Bertrand B. Schöpflin R. (2021). Non-coding deletions identify Maenli lncRNA as a limb-specific En1 regulator. Nature 592 (7852), 93–98. 10.1038/s41586-021-03208-9 33568816

[B7] Amelio I. Bernassola F. Candi E. (2021). Emerging roles of long non-coding RNAs in breast cancer biology and management. Seminars Cancer Biol. 72, 36–45. 10.1016/j.semcancer.2020.06.019 32619506

[B8] Augoff K. McCue B. Plow E. F. Sossey-Alaoui K. (2012). miR-31 and its host gene lncRNA LOC554202 are regulated by promoter hypermethylation in triple-negative breast cancer. Mol. Cancer 11, 5. 10.1186/1476-4598-11-5 22289355 PMC3298503

[B9] Badowski C. He B. Garmire L. X. (2022). Blood-derived lncRNAs as biomarkers for cancer diagnosis: the Good, the Bad and the Beauty. NPJ Precis. Oncol. 6 (1), 40. 10.1038/s41698-022-00283-7 35729321 PMC9213432

[B10] Basak P. Chatterjee S. Weger S. Bruce M. C. Murphy L. C. Raouf A. (2015). Estrogen regulates luminal progenitor cell differentiation through H19 gene expression. Endocrine-related cancer 22 (4), 505–517. 10.1530/ERC-15-0105 25944846 PMC4498491

[B11] Bassett A. R. Akhtar A. Barlow D. P. Bird A. P. Brockdorff N. Duboule D. (2014). Considerations when investigating lncRNA function *in vivo* . eLife 3, e03058. 10.7554/eLife.03058 25124674 PMC4132285

[B12] Batista P. J. Chang H. Y. (2013). Long noncoding RNAs: cellular address codes in development and disease. Cell 152 (6), 1298–1307. 10.1016/j.cell.2013.02.012 23498938 PMC3651923

[B13] Beckedorff F. C. Ayupe A. C. Crocci-Souza R. Amaral M. S. Nakaya H. I. Soltys D. T. (2013). The intronic long noncoding RNA ANRASSF1 recruits PRC2 to the RASSF1A promoter, reducing the expression of RASSF1A and increasing cell proliferation. PLoS Genet. 9 (8), e1003705. 10.1371/journal.pgen.1003705 23990798 PMC3749938

[B14] Biondini M. Lehuédé C. Tabariès S. Annis M. G. Pacis A. Ma E. H. (2024). Metastatic breast cancer cells are metabolically reprogrammed to maintain redox homeostasis during metastasis. Redox Biol. 75, 103276. 10.1016/j.redox.2024.103276 39053265 PMC11321393

[B15] Biswas S. K. Allavena P. Mantovani A. (2013). Tumor-associated macrophages: functional diversity, clinical significance, and open questions. Seminars Immunopathol. 35 (5), 585–600. 10.1007/s00281-013-0367-7 23657835

[B16] Boretto C. Actis C. Faris P. Cordero F. Beccuti M. Ferrero G. (2023). Tamoxifen activates transcription factor EB and triggers protective autophagy in breast cancer cells by inducing lysosomal calcium release: a gateway to the onset of endocrine resistance. Int. J. Mol. Sci. 25 (1), 458. 10.3390/ijms25010458 38203629 PMC10779225

[B17] Bray F. Laversanne M. Sung H. Ferlay J. Siegel R. L. Soerjomataram I. (2024). Global cancer statistics 2022: GLOBOCAN estimates of incidence and mortality worldwide for 36 cancers in 185 countries. CA Cancer J. Clin. 74 (3), 229–263. 10.3322/caac.21834 38572751

[B18] Bury M. Le Calvé B. Ferbeyre G. Blank V. Lessard F. (2021). New insights into CDK regulators: novel opportunities for cancer therapy. Trends cell Biol. 31 (5), 331–344. 10.1016/j.tcb.2021.01.010 33676803

[B19] Cabili M. N. Trapnell C. Goff L. Koziol M. Tazon-Vega B. Regev A. (2011). Integrative annotation of human large intergenic noncoding RNAs reveals global properties and specific subclasses. Genes and Dev. 25 (18), 1915–1927. 10.1101/gad.17446611 21890647 PMC3185964

[B20] Cai Z. Shi Q. Li Y. Jin L. Li S. Wong L. L. (2023). LncRNA EILA promotes CDK4/6 inhibitor resistance in breast cancer by stabilizing cyclin E1 protein. Sci. Adv. 9 (40), eadi3821. 10.1126/sciadv.adi3821 37801505 PMC10558131

[B21] Caldon C. E. Sergio C. M. Kang J. Muthukaruppan A. Boersma M. N. Stone A. (2012). Cyclin E2 overexpression is associated with endocrine resistance but not insensitivity to CDK2 inhibition in human breast cancer cells. Mol. cancer Ther. 11 (7), 1488–1499. 10.1158/1535-7163.MCT-11-0963 22564725

[B22] Cao C. Li A. Xu C. Wu B. Yao L. Liu Y. (2024). Engineering artificial non-coding RNAs for targeted protein degradation. Nat. Chem. Biol. 21, 393–401. 10.1038/s41589-024-01719-w 39215101

[B23] Chan S. H. Tsai K. W. Chiu S. Y. Kuo W. H. Chen H. Y. Jiang S. S. (2019). Identification of the novel role of CD24 as an oncogenesis regulator and therapeutic target for triple-negative breast cancer. Mol. cancer Ther. 18 (1), 147–161. 10.1158/1535-7163.MCT-18-0292 30381446

[B24] Chen D. Washbrook E. Sarwar N. Bates G. J. Pace P. E. Thirunuvakkarasu V. (2002). Phosphorylation of human estrogen receptor alpha at serine 118 by two distinct signal transduction pathways revealed by phosphorylation-specific antisera. Oncogene 21 (32), 4921–4931. 10.1038/sj.onc.1205420 12118371

[B25] Chen J. Wang Y. Wang C. Hu J. F. Li W. (2020). LncRNA functions as a new emerging epigenetic factor in determining the fate of stem cells. Front. Genet. 11, 277. 10.3389/fgene.2020.00277 32296461 PMC7137347

[B26] Chen L. L. (2016). Linking long noncoding RNA localization and function. Trends Biochem. Sci. 41 (9), 761–772. 10.1016/j.tibs.2016.07.003 27499234

[B27] Chen Q. F. Hu C. F. Sun K. Lang Y. P. (2019). LncRNA NR027113 promotes malignant progression of gastric carcinoma via EMT signaling pathway. Eur. Rev. Med. Pharmacol. Sci. 23 (11), 4746–4755. 10.26355/eurrev_201906_18056 31210301

[B28] Chen S. Lai S. W. T. Brown C. E. Feng M. (2021b). Harnessing and enhancing macrophage phagocytosis for cancer therapy. Front. Immunol. 12, 635173. 10.3389/fimmu.2021.635173 33790906 PMC8006289

[B29] Chen Y. Li Z. Chen X. Zhang S. (2021a). Long non-coding RNAs: from disease code to drug role. Acta Pharm. Sin. B 11 (2), 340–354. 10.1016/j.apsb.2020.10.001 33643816 PMC7893121

[B30] Chen Z. Tian D. Chen X. Cheng M. Xie H. Zhao J. (2023). Super-enhancer-driven lncRNA LIMD1-AS1 activated by CDK7 promotes glioma progression. Cell death and Dis. 14 (6), 383. 10.1038/s41419-023-05892-z PMC10310775 37385987

[B31] Chen Z. Zhou Z. Y. He C. C. Zhang J. L. Wang J. Xiao Z. Y. (2018). Down-regulation of LncRNA NR027113 inhibits cell proliferation and metastasis via PTEN/PI3K/AKT signaling pathway in hepatocellular carcinoma. Eur. Rev. Med. Pharmacol. Sci. 22 (21), 7222–7232. 10.26355/eurrev_201811_16256 30468465

[B32] Chou J. Quigley D. A. Robinson T. M. Feng F. Y. Ashworth A. (2020). Transcription-associated cyclin-dependent kinases as targets and biomarkers for cancer therapy. Cancer Discov. 10 (3), 351–370. 10.1158/2159-8290.CD-19-0528 32071145

[B33] Christofk H. R. Vander Heiden M. G. Harris M. H. Ramanathan A. Gerszten R. E. Wei R. (2008). The M2 splice isoform of pyruvate kinase is important for cancer metabolism and tumour growth. Nature 452 (7184), 230–233. 10.1038/nature06734 18337823

[B34] Chu C. Geng Y. Zhou Y. Sicinski P. (2021). Cyclin E in normal physiology and disease states. Trends cell Biol. 31 (9), 732–746. 10.1016/j.tcb.2021.05.001 34052101 PMC8364501

[B35] Chu W. Zhang X. Qi L. Fu Y. Wang P. Zhao W. (2020). The EZH2-PHACTR2-AS1-ribosome Axis induces genomic instability and promotes growth and metastasis in breast cancer. Cancer Res. 80 (13), 2737–2750. 10.1158/0008-5472.CAN-19-3326 32312833

[B36] Cortés-Cros M. Hemmerlin C. Ferretti S. Zhang J. Gounarides J. S. Yin H. (2013). M2 isoform of pyruvate kinase is dispensable for tumor maintenance and growth. Proc. Natl. Acad. Sci. U. S. A. 110 (2), 489–494. 10.1073/pnas.1212780110 23267074 PMC3545759

[B37] Cui K. Wu H. Zhang L. Li H. Israr G. Li Z. (2023). A novel lncRNA 495810 promotes the aerobic glycolysis in colorectal cancer by stabilizing pyruvate kinase isozyme M2. Int. J. Oncol. 62 (5), 58. 10.3892/ijo.2023.5506 36960860

[B38] Cui M. Liu Y. Cui L. (2022). Long non-coding RNA LINC02613 is a prognostic biomarker for breast cancer and correlates with the cell cycle and immune infiltration based on TCGA data. Transl. cancer Res. 11 (4), 615–628. 10.21037/tcr-21-2479 35571659 PMC9091017

[B39] Das C. K. Parekh A. Parida P. K. Bhutia S. K. Mandal M. (2019). Lactate dehydrogenase A regulates autophagy and tamoxifen resistance in breast cancer. Biochimica biophysica acta Mol. cell Res. 1866 (6), 1004–1018. 10.1016/j.bbamcr.2019.03.004 30878502

[B40] Debnath J. Gammoh N. Ryan K. M. (2023). Autophagy and autophagy-related pathways in cancer. Nat. Rev. Mol. cell Biol. 24 (8), 560–575. 10.1038/s41580-023-00585-z 36864290 PMC9980873

[B41] Deng J. Wang E. S. Jenkins R. W. Li S. Dries R. Yates K. (2018). CDK4/6 inhibition augments antitumor immunity by enhancing T-cell activation. Cancer Discov. 8 (2), 216–233. 10.1158/2159-8290.CD-17-0915 29101163 PMC5809273

[B42] Derrien T. Johnson R. Bussotti G. Tanzer A. Djebali S. Tilgner H. (2012). The GENCODE v7 catalog of human long noncoding RNAs: analysis of their gene structure, evolution, and expression. Genome Res. 22 (9), 1775–1789. 10.1101/gr.132159.111 22955988 PMC3431493

[B43] Devanaboyina M. Kaur J. Whiteley E. Lin L. Einloth K. Morand S. (2022). NF-κB signaling in tumor pathways focusing on breast and ovarian cancer. Oncol. Rev. 16, 10568. 10.3389/or.2022.10568 36531159 PMC9756851

[B44] Dong F. Ruan S. Wang J. Xia Y. Le K. Xiao X. (2020). M2 macrophage-induced lncRNA PCAT6 facilitates tumorigenesis and angiogenesis of triple-negative breast cancer through modulation of VEGFR2. Cell death and Dis. 11 (9), 728. 10.1038/s41419-020-02926-8 PMC7481779 32908134

[B45] Dou D. R. Zhao Y. Belk J. A. Zhao Y. Casey K. M. Chen D. C. (2024). Xist ribonucleoproteins promote female sex-biased autoimmunity. Cell. 187 (3), 733–749.e16. 10.1016/j.cell.2023.12.037 38306984 PMC10949934

[B46] Dupuy F. Tabariès S. Andrzejewski S. Dong Z. Blagih J. Annis M. G. (2015). PDK1-Dependent metabolic reprogramming dictates metastatic potential in breast cancer. Cell metab. 22 (4), 577–589. 10.1016/j.cmet.2015.08.007 26365179

[B47] Economopoulou P. Kotsantis I. Psyrri A. (2020). Tumor microenvironment and immunotherapy response in head and neck cancer. Cancers 12 (11), 3377. 10.3390/cancers12113377 33203092 PMC7696050

[B48] Eissa S. Safwat M. Matboli M. Zaghloul A. El-Sawalhi M. Shaheen A. (2019). Measurement of urinary level of a specific competing endogenous RNA network (FOS and RCAN mRNA/miR-324-5p, miR-4738-3p, /lncRNA miR-497-HG) enables diagnosis of bladder cancer. Urol. Oncol. 37 (4), 292.e19–292.e27. 10.1016/j.urolonc.2018.12.024 30654976

[B49] ENCODE Project Consortium (2012). An integrated encyclopedia of DNA elements in the human genome. Nature 489 (7414), 57–74. 10.1038/nature11247 22955616 PMC3439153

[B50] Fei M. Li X. Liang S. Zhou S. Wu H. Sun L. (2024). LncRNA PWRN1 inhibits the progression of hepatocellular carcinoma by activating PKM2 activity. Cancer Lett. 584, 216620. 10.1016/j.canlet.2024.216620 38218456

[B51] Ferrer J. Dimitrova N. (2024). Transcription regulation by long non-coding RNAs: mechanisms and disease relevance. Nat. Rev. Mol. cell Biol. 25 (5), 396–415. 10.1038/s41580-023-00694-9 38242953 PMC11045326

[B52] Fong M. Y. Zhou W. Liu L. Alontaga A. Y. Chandra M. Ashby J. (2015). Breast-cancer-secreted miR-122 reprograms glucose metabolism in premetastatic niche to promote metastasis. Nat. cell Biol. 17 (2), 183–194. 10.1038/ncb3094 25621950 PMC4380143

[B53] Fu M. Wang C. Li Z. Sakamaki T. Pestell R. G. (2004). Minireview: cyclin D1: normal and abnormal functions. Endocrinology 145 (12), 5439–5447. 10.1210/en.2004-0959 15331580

[B54] Fu Y. Zhang X. Liu X. Wang P. Chu W. Zhao W. (2022). The DNMT1-PAS1-PH20 axis drives breast cancer growth and metastasis. Signal Transduct. Target. Ther. 7 (1), 81. 10.1038/s41392-022-00896-1 35307730 PMC8934873

[B55] Ganesh V. S. Riquin K. Chatron N. Yoon E. Lamar K. M. Aziz M. C. (2024). Neurodevelopmental disorder caused by deletion of CHASERR, a lncRNA gene. N. Engl. J. Med. 391 (16), 1511–1518. 10.1056/NEJMoa2400718 39442041 PMC11826417

[B56] Geisler S. Coller J. (2013). RNA in unexpected places: long non-coding RNA functions in diverse cellular contexts. Nat. Rev. Mol. cell Biol. 14 (11), 699–712. 10.1038/nrm3679 24105322 PMC4852478

[B57] Giaquinto A. N. Sung H. Miller K. D. Kramer J. L. Newman L. A. Minihan A. (2022). Breast cancer statistics, 2022. CA Cancer J. Clin. 72 (6), 524–541. 10.3322/caac.21754 36190501

[B58] Goel S. DeCristo M. J. Watt A. C. BrinJones H. Sceneay J. Li B. B. (2017). CDK4/6 inhibition triggers anti-tumour immunity. Nature 548 (7668), 471–475. 10.1038/nature23465 28813415 PMC5570667

[B59] Goyal B. Yadav S. R. M. Awasthee N. Gupta S. Kunnumakkara A. B. Gupta S. C. (2021). Diagnostic, prognostic, and therapeutic significance of long non-coding RNA MALAT1 in cancer. Biochimica biophysica acta Rev. Cancer 1875 (2), 188502. 10.1016/j.bbcan.2021.188502 33428963

[B60] Gui D. Y. Lewis C. A. Vander Heiden M. G. (2013). Allosteric regulation of PKM2 allows cellular adaptation to different physiological states. Sci. Signal. 6 (263), pe7. 10.1126/scisignal.2003925 23423437

[B61] Guiducci G. Stojic L. (2021). Long noncoding RNAs at the crossroads of cell cycle and genome integrity. Trends Genet. TIG. 37 (6), 528–546. 10.1016/j.tig.2021.01.006 33685661

[B62] Guiley K. Z. Stevenson J. W. Lou K. Barkovich K. J. Kumarasamy V. Wijeratne T. U. (2019). p27 allosterically activates cyclin-dependent kinase 4 and antagonizes palbociclib inhibition. Science 366 (6471), eaaw2106. 10.1126/science.aaw2106 31831640 PMC7592119

[B63] Guo R. Su Y. Zhang Q. Xiu B. Huang S. Chi W. (2023). LINC00478-derived novel cytoplasmic lncRNA LacRNA stabilizes PHB2 and suppresses breast cancer metastasis via repressing MYC targets. J. Transl. Med. 21 (1), 120. 10.1186/s12967-023-03967-1 36782197 PMC9926633

[B64] Guttman M. Amit I. Garber M. French C. Lin M. F. Feldser D. (2009). Chromatin signature reveals over a thousand highly conserved large non-coding RNAs in mammals. Nature 458 (7235), 223–227. 10.1038/nature07672 19182780 PMC2754849

[B65] Guzel E. Okyay T. M. Yalcinkaya B. Karacaoglu S. Gocmen M. Akcakuyu M. H. (2020). Tumor suppressor and oncogenic role of long non-coding RNAs in cancer. North. Clin. Istanbul 7 (1), 81–86. 10.14744/nci.2019.46873 PMC7103751 32232211

[B66] Hafner M. Mills C. E. Subramanian K. Chen C. Chung M. Boswell S. A. (2019). Multiomics profiling establishes the polypharmacology of FDA-approved CDK4/6 inhibitors and the potential for differential clinical activity. Cell Chem. Biol. 26 (8), 1067–1080.e8. 10.1016/j.chembiol.2019.05.005 31178407 PMC6936329

[B67] Hagemann T. Wilson J. Kulbe H. Li N. F Leinster D. A. Charles K. (2005). Macrophages induce invasiveness of epithelial cancer cells via NF-kappa B and JNK. J. Immunol. Baltim. 175 (2), 1197–1205. 10.4049/jimmunol.175.2.1197 16002723

[B68] Han M. Qian X. Cao H. Wang F. Li X. Han N. (2020). LncRNA ZNF649-AS1 induces trastuzumab resistance by promoting ATG5 expression and autophagy. Mol. Ther. J. Am. Soc. Gene Ther. 28 (11), 2488–2502. 10.1016/j.ymthe.2020.07.019 PMC7647669 32735773

[B69] Hanahan D. Weinberg R. A. (2011). Hallmarks of cancer: the next generation. Cell 144 (5), 646–674. 10.1016/j.cell.2011.02.013 21376230

[B70] He X. Dong K. Shen J. Hu G. Mintz J. D. Atawia R. T. (2023). The long noncoding RNA cardiac mesoderm enhancer-associated noncoding RNA (Carmn) is a critical regulator of gastrointestinal smooth muscle contractile function and motility. Gastroenterology 165 (1), 71–87. 10.1053/j.gastro.2023.03.229 37030336 PMC10330198

[B71] Herman A. B. Tsitsipatis D. Gorospe M. (2022). Integrated lncRNA function upon genomic and epigenomic regulation. Mol. Cell 82 (12), 2252–2266. 10.1016/j.molcel.2022.05.027 35714586 PMC9219586

[B72] Hiebert S. W. Chellappan S. P. Horowitz J. M. Nevins J. R. (1992). The interaction of RB with E2F coincides with an inhibition of the transcriptional activity of E2F. Genes and Dev. 6 (2), 177–185. 10.1101/gad.6.2.177 1531329

[B73] Huang S. L. Huang Z. C. Zhang C. J. Xie J. Lei S. S. Wu Y. Q. (2022). LncRNA SNHG5 promotes the glycolysis and proliferation of breast cancer cell through regulating BACH1 via targeting miR-299. Breast Cancer (Tokyo, Jpn.) 29 (1), 65–76. 10.1007/s12282-021-01281-6 PMC8732815 34351577

[B74] Huang X. J. Xia Y. He G. F. Zheng L. L. Cai Y. P. Yin Y. (2018). MALAT1 promotes angiogenesis of breast cancer. Oncol. Rep. 40 (5), 2683–2689. 10.3892/or.2018.6705 30226550

[B75] Hwang H. C. Clurman B. E. (2005). Cyclin E in normal and neoplastic cell cycles. Oncogene 24 (17), 2776–2786. 10.1038/sj.onc.1208613 15838514

[B76] Ishfaq M. Bashir N. Riaz S. K. Manzoor S. Khan J. S. Bibi Y. (2022). Expression of HK2, PKM2, and PFKM is associated with metastasis and late disease onset in breast cancer patients. Genes 13 (3), 549. 10.3390/genes13030549 35328104 PMC8955648

[B77] Israelsen W. J. Dayton T. L. Davidson S. M. Fiske B. P. Hosios A. M. Bellinger G. (2013). PKM2 isoform-specific deletion reveals a differential requirement for pyruvate kinase in tumor cells. Cell 155 (2), 397–409. 10.1016/j.cell.2013.09.025 24120138 PMC3850755

[B78] Jiang B. Liu Q. Gai J. Guan J. Li Q. (2022). LncRNA SLC16A1-AS1 regulates the miR-182/PDCD4 axis and inhibits the triple-negative breast cancer cell cycle. Immunopharmacol. Immunotoxicol. 44 (4), 534–540. 10.1080/08923973.2022.2056482 35316129

[B79] Jiang T. Zhu J. Jiang S. Chen Z. Xu P. Gong R. (2023). Targeting lncRNA DDIT4-AS1 sensitizes triple negative breast cancer to chemotherapy via suppressing of autophagy. Adv. Sci. (Weinheim, Baden-Wurttemberg, Ger.) 10 (17), e2207257. 10.1002/advs.202207257 PMC10265098 37096846

[B80] Jin X. Ge L. P. Li D. Q. Shao Z. M. Di G. H. Xu X. E. (2020). LncRNA TROJAN promotes proliferation and resistance to CDK4/6 inhibitor via CDK2 transcriptional activation in ER+ breast cancer. Mol. Cancer 19 (1), 87. 10.1186/s12943-020-01210-9 32393270 PMC7212688

[B81] Jin X. Xu X. E. Jiang Y. Z. Liu Y. R. Sun W. Guo Y. J. (2019). The endogenous retrovirus-derived long noncoding RNA TROJAN promotes triple-negative breast cancer progression via ZMYND8 degradation. Sci. Adv. 5 (3), eaat9820. 10.1126/sciadv.aat9820 30854423 PMC6402854

[B82] Khani-Habibabadi F. Zare L. Sahraian M. A. Javan M. Behmanesh M. (2022). Hotair and Malat1 long noncoding RNAs regulate bdnf expression and oligodendrocyte precursor cell differentiation. Mol. Neurobiol. 59 (7), 4209–4222. 10.1007/s12035-022-02844-0 35499794

[B83] Kim J. Piao H. L. Kim B. J. Yao F. Han Z. Wang Y. (2018). Long noncoding RNA MALAT1 suppresses breast cancer metastasis. Nat. Genet. 50 (12), 1705–1715. 10.1038/s41588-018-0252-3 30349115 PMC6265076

[B84] Kitagawa M. Kitagawa K. Kotake Y. Niida H. Ohhata T. (2013). Cell cycle regulation by long non-coding RNAs. Cell. Mol. Life Sci. CMLS 70 (24), 4785–4794. 10.1007/s00018-013-1423-0 23880895 PMC3830198

[B85] Klein M. E. Kovatcheva M. Davis L. E. Tap W. D. Koff A. (2018). CDK4/6 inhibitors: the mechanism of action may not Be as simple as once thought. Cancer cell 34 (1), 9–20. 10.1016/j.ccell.2018.03.023 29731395 PMC6039233

[B86] Kurisu S. Suetsugu S. Yamazaki D. Yamaguchi H. Takenawa T. (2005). Rac-WAVE2 signaling is involved in the invasive and metastatic phenotypes of murine melanoma cells. Oncogene 24 (8), 1309–1319. 10.1038/sj.onc.1208177 15608687

[B87] Lazorthes S. Vallot C. Briois S. Aguirrebengoa M. Thuret J. Y. St Laurent G. (2015). A vlincRNA participates in senescence maintenance by relieving H2AZ-mediated repression at the INK4 locus. Nat. Commun. 6, 5971. 10.1038/ncomms6971 25601475 PMC4309439

[B88] Leek R. D. Lewis C. E. Whitehouse R. Greenall M. Clarke J. Harris A. L. (1996). Association of macrophage infiltration with angiogenesis and prognosis in invasive breast carcinoma. Cancer Res. 56 (20), 4625–4629. 8840975

[B89] Levine B. Kroemer G. (2019). Biological functions of autophagy genes: a disease perspective. Cell 176 (1-2), 11–42. 10.1016/j.cell.2018.09.048 30633901 PMC6347410

[B90] Li J. Meng H. Bai Y. Wang K. (2016). Regulation of lncRNA and its role in cancer metastasis. Oncol. Res. 23 (5), 205–217. 10.3727/096504016x14549667334007 27098144 PMC7838649

[B91] Li P. P. Li R. G. Huang Y. Q. Lu J. P. Zhang W. J. Wang Z. Y. (2021c). LncRNA OTUD6B-AS1 promotes paclitaxel resistance in triple negative breast cancer by regulation of miR-26a-5p/MTDH pathway-mediated autophagy and genomic instability. Aging 13 (21), 24171–24191. 10.18632/aging.203672 34740994 PMC8610138

[B92] Li X. Ma T. K. Wang M. Zhang X. D. Liu T. Y. Liu Y. (2023). YY1-induced upregulation of LncRNA-ARAP1-AS2 and ARAP1 promotes diabetic kidney fibrosis via aberrant glycolysis associated with EGFR/PKM2/HIF-1α pathway. Front. Pharmacol. 14, 1069348. 10.3389/fphar.2023.1069348 36874012 PMC9974832

[B93] Li X. Zhang J. L. Lei Y. N. Liu X. Q. Xue W. Zhang Y. (2021a). Linking circular intronic RNA degradation and function in transcription by RNase H1. Sci. China Life Sci. 64 (11), 1795–1809. 10.1007/s11427-021-1993-6 34453665

[B94] Li Y. Cook K. L. Yu W. Jin L. Bouker K. B. Clarke R. (2021b). Inhibition of antiestrogen-promoted pro-survival autophagy and tamoxifen resistance in breast cancer through vitamin D receptor. Nutrients 13 (5), 1715. 10.3390/nu13051715 34069442 PMC8159129

[B95] Li Z. Qian J. Li J. Zhu C. (2019). Knockdown of lncRNA-HOTAIR downregulates the drug-resistance of breast cancer cells to doxorubicin via the PI3K/AKT/mTOR signaling pathway. Exp. Ther. Med. 18 (1), 435–442. 10.3892/etm.2019.7629 31281438 PMC6580102

[B96] Li Z. M. Liu G. Gao Y. Zhao M. G. (2022). Targeting CDK7 in oncology: the avenue forward. Pharmacol. and Ther. 240, 108229. 10.1016/j.pharmthera.2022.108229 35700828

[B97] Liang X. H. Jackson S. Seaman M. Brown K. Kempkes B. Hibshoosh H. (1999). Induction of autophagy and inhibition of tumorigenesis by beclin 1. Nature 402 (6762), 672–676. 10.1038/45257 10604474

[B98] Lin A. Giuliano C. J. Sayles N. M. Sheltzer J. M. (2017). CRISPR/Cas9 mutagenesis invalidates a putative cancer dependency targeted in on-going clinical trials. eLife 6, e24179. 10.7554/eLife.24179 28337968 PMC5365317

[B99] Lin N. U. Winer E. P. (2007). Brain metastases: the HER2 paradigm. Clin. cancer Res. official J. Am. Assoc. Cancer Res. 13 (6), 1648–1655. 10.1158/1078-0432.CCR-06-2478 17363517

[B100] Liu B. Qu X. Wang J. Xu L. Zhang L. Xu B. (2023b). LINC00365 functions as a tumor suppressor by inhibiting HIF-1α-mediated glucose metabolism reprogramming in breast cancer. Exp. cell Res. 425 (1), 113514. 10.1016/j.yexcr.2023.113514 36804531

[B101] Liu J. Lao L. Chen J. Li J. Zeng W. Zhu X. (2021b). The IRENA lncRNA converts chemotherapy-polarized tumor-suppressing macrophages to tumor-promoting phenotypes in breast cancer. Nat. Cancer 2 (4), 457–473. 10.1038/s43018-021-00196-7 35122000

[B102] Liu J. Shi Y. Wu M. Zhang F. Xu M. He Z. (2023a). JAG1 enhances angiogenesis in triple-negative breast cancer through promoting the secretion of exosomal lncRNA MALAT1. Genes and Dis. 10 (5), 2167–2178. 10.1016/j.gendis.2022.07.006 PMC10363582 37492742

[B103] Liu J. Yan C. Xu S. (2024). LncRNA IL21-AS1 facilitates tumour progression by enhancing CD24-induced phagocytosis inhibition and tumorigenesis in ovarian cancer. Cell death and Dis. 15 (5), 313. 10.1038/s41419-024-06704-8 PMC11068771 38702326

[B104] Liu S. J. Dang H. X. Lim D. A. Feng F. Y. Maher C. A. (2021a). Long noncoding RNAs in cancer metastasis. Nat. Rev. Cancer 21 (7), 446–460. 10.1038/s41568-021-00353-1 33953369 PMC8288800

[B105] Liu X. Zhang G. Yu T. Liu J. Chai X. Yin D. (2023d). CL4-modified exosomes deliver lncRNA DARS-AS1 siRNA to suppress triple-negative breast cancer progression and attenuate doxorubicin resistance by inhibiting autophagy. Int. J. Biol. Macromol. 250, 126147. 10.1016/j.ijbiomac.2023.126147 37544559

[B106] Liu Y. Shi M. He X. Cao Y. Liu P. Li F. (2022). LncRNA-PACERR induces pro-tumour macrophages via interacting with miR-671-3p and m6A-reader IGF2BP2 in pancreatic ductal adenocarcinoma. J. Hematol. and Oncol. 15 (1), 52. 10.1186/s13045-022-01272-w 35526050 PMC9077921

[B107] Liu Y. M. Ge J. Y. Chen Y. F. Liu T. Chen L. Liu C. C. (2023c). Combined single-cell and spatial transcriptomics reveal the metabolic evolvement of breast cancer during early dissemination. Adv. Sci. (Weinheim, Baden-Wurttemberg, Ger.) 10 (6), e2205395. 10.1002/advs.202205395 PMC9951304 36594618

[B108] Luo Q. Shen F. Zhao S. Dong L. Wei J. Hu H. (2024). LINC00460/miR-186-3p/MYC feedback loop facilitates colorectal cancer immune escape by enhancing CD47 and PD-L1 expressions. J. Exp. and Clin. Cancer Res. CR 43 (1), 225. 10.1186/s13046-024-03145-1 39135122 PMC11321182

[B109] Lynce F. Shajahan-Haq A. N. Swain S. M. (2018). CDK_4_/6 inhibitors in breast cancer therapy: current practice and future opportunities. Pharmacol. and Ther. 191, 65–73. 10.1016/j.pharmthera.2018.06.008 29933034 PMC6533626

[B110] Malumbres M. Barbacid M. (2009). Cell cycle, CDKs and cancer: a changing paradigm. Nat. Rev. Cancer 9 (3), 153–166. 10.1038/nrc2602 19238148

[B111] Mao C. Wang X. Liu Y. Wang M. Yan B. Jiang Y. (2018). A G3BP1-interacting lncRNA promotes ferroptosis and apoptosis in cancer via nuclear sequestration of p53. Cancer Res. 78 (13), 3484–3496. 10.1158/0008-5472.CAN-17-3454 29588351 PMC8073197

[B112] Marino N. Woditschka S. Reed L. T. Nakayama J. Mayer M. Wetzel M. (2013). Breast cancer metastasis: issues for the personalization of its prevention and treatment. Am. J. Pathol. 183 (4), 1084–1095. 10.1016/j.ajpath.2013.06.012 23895915 PMC3791679

[B113] Meng Z. Zhang R. Wu X. Piao Z. Zhang M. Jin T. (2024). LncRNA HAGLROS promotes breast cancer evolution through miR-135b-3p/COL10A1 axis and exosome-mediated macrophage M2 polarization. Cell Death and Dis. 15 (8), 633. 10.1038/s41419-024-07020-x PMC11358487 39198393

[B114] Mercer T. R. Dinger M. E. Sunkin S. M. Mehler M. F. Mattick J. S. (2008). Specific expression of long noncoding RNAs in the mouse brain. Proc. Natl. Acad. Sci. U. S. A. 105 (2), 716–721. 10.1073/pnas.0706729105 18184812 PMC2206602

[B115] Miao C. Bai L. Yang Y. Huang J. (2021). Dysregulation of lncRNAs in rheumatoid arthritis: biomarkers, pathogenesis and potential therapeutic targets. Front. Pharmacol. 12, 652751. 10.3389/fphar.2021.652751 33776780 PMC7994855

[B116] Mondal P. Meeran S. M. (2020). Long non-coding RNAs in breast cancer metastasis. Non-Coding RNA Res. 5 (4), 208–218. 10.1016/j.ncrna.2020.11.004 PMC7689374 33294746

[B117] Morrison L. Loibl S. Turner N. C. (2024). The CDK4/6 inhibitor revolution - a game-changing era for breast cancer treatment. Nat. Rev. Clin. Oncol. 21 (2), 89–105. 10.1038/s41571-023-00840-4 38082107

[B118] Murray P. J. Allen J. E. Biswas S. K. Fisher E. A. Gilroy D. W. Goerdt S. (2014). Macrophage activation and polarization: nomenclature and experimental guidelines. Immunity 41 (1), 14–20. 10.1016/j.immuni.2014.06.008 25035950 PMC4123412

[B119] Nguyen D. X. Bos P. D. Massagué J. (2009). Metastasis: from dissemination to organ-specific colonization. Nat. Rev. Cancer 9 (4), 274–284. 10.1038/nrc2622 19308067

[B120] Ouyang J. Liu Z. Yuan X. Long C. Chen X. Wang Y. (2021). LncRNA PRNCR1 promotes breast cancer proliferation and inhibits apoptosis by modulating microRNA-377/CCND2/MEK/MAPK Axis. Archives Med. Res. 52 (5), 471–482. 10.1016/j.arcmed.2021.01.007 33608112

[B121] Palsson-McDermott E. M. Curtis A. M. Goel G. Lauterbach M. A. R. Sheedy F. J. Gleeson L. E. (2015). Pyruvate kinase M2 regulates hif-1α activity and IL-1β induction and is a critical determinant of the warburg effect in LPS-activated macrophages. Cell metab. 21 (2), 65–80. 10.1016/j.cmet.2014.12.005 25565206 PMC5198835

[B122] Pandey K. An H. J. Kim S. K. Lee S. A. Kim S. Lim S. M. (2019). Molecular mechanisms of resistance to CDK_4_/6 inhibitors in breast cancer: a review. Int. J. cancer 145 (5), 1179–1188. 10.1002/ijc.32020 30478914 PMC6767051

[B123] Pang D. Hu Q. Lan X. Lin Y. Duan H. Cao S. (2019). The novel long non-coding RNA PRNCR1-2 is involved in breast cancer cell proliferation, migration, invasion and cell cycle progression. Mol. Med. Rep. 19 (3), 1824–1832. 10.3892/mmr.2018.9789 30592261

[B124] Pavletich N. P. (1999). Mechanisms of cyclin-dependent kinase regulation: structures of Cdks, their cyclin activators, and Cip and INK4 inhibitors. J. Mol. Biol. 287 (5), 821–828. 10.1006/jmbi.1999.2640 10222191

[B125] Peralta-Alvarez C. A. Núñez-Martínez H. N. Cerecedo-Castillo Á. J. Poot-Hernández A. C. Tapia-Urzúa G. Garza-Manero S. (2024). A bidirectional non-coding RNA promoter mediates long-range gene expression regulation. Genes 15 (5), 549. 10.3390/genes15050549 38790178 PMC11120797

[B126] Qian B. Z. Li J. Zhang H. Kitamura T. Zhang J. Campion L. R. (2011). CCL_2_ recruits inflammatory monocytes to facilitate breast-tumour metastasis. Nature 475 (7355), 222–225. 10.1038/nature10138 21654748 PMC3208506

[B127] Qian X. Zhao J. Yeung P. Y. Zhang Q. C. Kwok C. K. (2019). Revealing lncRNA structures and interactions by sequencing-based approaches. Trends Biochem. Sci. 44 (1), 33–52. 10.1016/j.tibs.2018.09.012 30459069

[B128] Quinn J. J. Chang H. Y. (2016). Unique features of long non-coding RNA biogenesis and function. Nat. Rev. Genet. 17 (1), 47–62. 10.1038/nrg.2015.10 26666209

[B129] Rugo H. S. Lerebours F. Ciruelos E. Drullinsky P. Ruiz-Borrego M. Neven P. (2021). Alpelisib plus fulvestrant in PIK3CA-mutated, hormone receptor-positive advanced breast cancer after a CDK4/6 inhibitor (BYLieve): one cohort of a phase 2, multicentre, open-label, non-comparative study. Lancet Oncol. 22 (4), 489–498. 10.1016/S1470-2045(21)00034-6 33794206

[B130] Rybstein M. D. Bravo-San Pedro J. M. Kroemer G. Galluzzi L. (2018). The autophagic network and cancer. Nat. cell Biol. 20 (3), 243–251. 10.1038/s41556-018-0042-2 29476153

[B131] Samir A. Tawab R. A. El Tayebi H. M. (2021). Long non-coding RNAs XIST and MALAT1 hijack the PD-L1 regulatory signaling pathway in breast cancer subtypes. Oncol. Lett. 22 (2), 593. 10.3892/ol.2021.12854 34149904 PMC8200942

[B132] Sava G. P. Fan H. Coombes R. C. Buluwela L. Ali S. (2020). CDK7 inhibitors as anticancer drugs. Cancer metastasis Rev. 39 (3), 805–823. 10.1007/s10555-020-09885-8 32385714 PMC7497306

[B133] Scaltriti M. Eichhorn P. J. Cortés J. Prudkin L. Aura C. Jiménez J. (2011). Cyclin E amplification/overexpression is a mechanism of trastuzumab resistance in HER2+ breast cancer patients. Proc. Natl. Acad. Sci. U. S. A. 108 (9), 3761–3766. 10.1073/pnas.1014835108 21321214 PMC3048107

[B134] Schito L. Rey S. (2017). Hypoxic pathobiology of breast cancer metastasis. Biochimica Biophysica Acta Rev. Cancer 1868 (1), 239–245. 10.1016/j.bbcan.2017.05.004 28526262

[B135] Sellers W. R. Rodgers J. W. Kaelin W. G. Jr (1995). A potent transrepression domain in the retinoblastoma protein induces a cell cycle arrest when bound to E2F sites. Proc. Natl. Acad. Sci. U. S. A. 92 (25), 11544–11548. 10.1073/pnas.92.25.11544 8524800 PMC40438

[B136] Shao J. Zhang Q. Wang P. Wang Z. (2021). LncRNA MALAT1 promotes breast cancer progression by sponging miR101-3p to mediate mTOR/PKM2 signal transmission. Am. J. Transl. Res. 13 (9), 10262–10275. 34650695 PMC8507063

[B137] Sharmin Z. Jin K. Gong A. Y. Deng S. Pok C. Graham M. L. (2024). LncRNA Nostrill promotes interferon-γ-stimulated gene transcription and facilitates intestinal epithelial cell-intrinsic anti-Cryptosporidium defense. Front. Immunol. 15, 1397117. 10.3389/fimmu.2024.1397117 39040107 PMC11260782

[B138] Shen Q. Sun Y. Xu S. (2020). LINC01503/miR-342-3p facilitates malignancy in non-small-cell lung cancer cells via regulating LASP1. Respir. Res. 21 (1), 235. 10.1186/s12931-020-01464-3 32938459 PMC7493870

[B139] Shi Q. Li Y. Li S. Jin L. Lai H. Wu Y. (2020). LncRNA DILA1 inhibits Cyclin D1 degradation and contributes to tamoxifen resistance in breast cancer. Nat. Commun. 11 (1), 5513. 10.1038/s41467-020-19349-w 33139730 PMC7608661

[B140] Shi Y. Lu J. Zhou J. Tan X. He Y. Ding J. (2014). Long non-coding RNA Loc554202 regulates proliferation and migration in breast cancer cells. Biochem. biophysical Res. Commun. 446 (2), 448–453. 10.1016/j.bbrc.2014.02.144 24631686

[B141] Shipitsin M. Campbell L. L. Argani P. Weremowicz S. Bloushtain-Qimron N. Yao J. (2007). Molecular definition of breast tumor heterogeneity. Cancer Cell 11 (3), 259–273. 10.1016/j.ccr.2007.01.013 17349583

[B142] Si Z. Yu L. Jing H. Wu L. Wang X. (2021). Oncogenic lncRNA ZNF561-AS1 is essential for colorectal cancer proliferation and survival through regulation of miR-26a-3p/miR-128-5p-SRSF6 axis. J. Exp. and Clin. cancer Res. CR 40 (1), 78. 10.1186/s13046-021-01882-1 33622363 PMC7903733

[B143] Siddique R. Thangavelu L. S. R. Almalki W. H. Kazmi I. Kumar A. Mahajan S. (2024). lncRNAs and cyclin-dependent kinases: unveiling their critical roles in cancer progression. Pathology, Res. Pract. 258, 155333. 10.1016/j.prp.2024.155333 38723325

[B144] Singh D. Assaraf Y. G. Gacche R. N. (2022a). Long non-coding RNA mediated drug resistance in breast cancer. Drug Resist Updat 63, 100851. 10.1016/j.drup.2022.100851 35810716

[B145] Singh S. Shyamal S. Panda A. C. (2022b). Detecting RNA-RNA interactome. Wiley Interdiscip. Rev. RNA 13 (5), e1715. 10.1002/wrna.1715 35132791

[B146] Song X. Fang C. Dai Y. Sun Y. Qiu C. Lin X. (2024). Cyclin-dependent kinase 7 (CDK7) inhibitors as a novel therapeutic strategy for different molecular types of breast cancer. Br. J. Cancer 130 (8), 1239–1248. 10.1038/s41416-024-02589-8 38355840 PMC11014910

[B147] Sossey-Alaoui K. Safina A. Li X. Vaughan M. M. Hicks D. G. Bakin A. V. (2007). Down-regulation of WAVE3, a metastasis promoter gene, inhibits invasion and metastasis of breast cancer cells. Am. J. Pathol. 170 (6), 2112–2121. 10.2353/ajpath.2007.060975 17525277 PMC1899429

[B148] Statello L. Guo C. J. Chen L. L. Huarte M. (2021). Gene regulation by long non-coding RNAs and its biological functions. Nat. Rev. Mol. cell Biol. 22 (2), 96–118. 10.1038/s41580-020-00315-9 33353982 PMC7754182

[B149] Steeg P. S. (2006). Tumor metastasis: mechanistic insights and clinical challenges. Nat. Med. 12 (8), 895–904. 10.1038/nm1469 16892035

[B150] Swain S. M. Shastry M. Hamilton E. (2023). Targeting HER2-positive breast cancer: advances and future directions. Nat. Rev. Drug Discov. 22 (2), 101–126. 10.1038/s41573-022-00579-0 36344672 PMC9640784

[B151] Tao S. Chen Q. Lin C. Dong H. (2020). Linc00514 promotes breast cancer metastasis and M2 polarization of tumor-associated macrophages via Jagged1-mediated notch signaling pathway. J. Exp. and Clin. cancer Res. CR 39 (1), 191. 10.1186/s13046-020-01676-x 32943090 PMC7500027

[B152] Tian Y. Chen Z. H. Wu P. Zhang D. Ma Y. Liu X. F. (2023). MIR497HG-Derived miR-195 and miR-497 mediate tamoxifen resistance via PI3K/AKT signaling in breast cancer. Adv. Sci. (Weinheim, Baden-Wurttemberg, Ger.) 10 (12), e2204819. 10.1002/advs.202204819 PMC10131819 36815359

[B153] Torre L. A. Bray F. Siegel R. L. Ferlay J. Lortet-Tieulent J. Jemal A. (2015). Global cancer statistics, 2012. CA Cancer J. Clin. 65 (2), 87–108. 10.3322/caac.21262 25651787

[B154] Tsagakis I. Douka K. Birds I. Aspden J. L. (2020). Long non-coding RNAs in development and disease: conservation to mechanisms. J. Pathology 250 (5), 480–495. 10.1002/path.5405 PMC8638664 32100288

[B155] Tu B. Song K. Zhou Y. Sun H. Liu Z. Y. Lin L. C. (2023). METTL3 boosts mitochondrial fission and induces cardiac fibrosis by enhancing LncRNA GAS5 methylation. Pharmacol. Res. 194, 106840. 10.1016/j.phrs.2023.106840 37379961

[B156] Turner N. C. Liu Y. Zhu Z. Loi S. Colleoni M. Loibl S. (2019). Cyclin E1 expression and palbociclib efficacy in previously treated hormone receptor-positive metastatic breast cancer. J. Clin. Oncol. Official J. Am. Soc. Clin. Oncol. 37 (14), 1169–1178. 10.1200/JCO.18.00925 PMC6506420 30807234

[B157] Van den Bossche J. O'Neill L. A. Menon D. (2017). Macrophage immunometabolism: where are we (going)? Trends Immunol. 38 (6), 395–406. 10.1016/j.it.2017.03.001 28396078

[B158] Wagner J. Rapsomaniki M. A. Chevrier S. Anzeneder T. Langwieder C. Dykgers A. (2019). A single-cell atlas of the tumor and immune ecosystem of human breast cancer. Cell 177 (5), 1330–1345.e18. 10.1016/j.cell.2019.03.005 30982598 PMC6526772

[B159] Wang J. Xie S. Yang J. Xiong H. Jia Y. Zhou Y. (2019). The long noncoding RNA H19 promotes tamoxifen resistance in breast cancer via autophagy. J. Hematol. and Oncol. 12 (1), 81. 10.1186/s13045-019-0747-0 31340867 PMC6657081

[B160] Wang Q. Y. Peng L. Chen Y. Liao L. D. Chen J. X. Li M. (2020). Characterization of super-enhancer-associated functional lncRNAs acting as ceRNAs in ESCC. Mol. Oncol. 14 (9), 2203–2230. 10.1002/1878-0261.12726 32460441 PMC7463357

[B161] Wang X. Jian Q. Zhang Z. Gu J. Wang X. Wang Y. (2024). Effect of tumor-derived extracellular vesicle-shuttled lncRNA MALAT1 on proliferation, invasion and metastasis of triple-negative breast cancer by regulating macrophage M2 polarization via the POSTN/Hippo/YAP axis. Transl. Oncol. 49, 102076. 10.1016/j.tranon.2024.102076 39222611 PMC11402314

[B162] Wang Y. Fu Y. Lu Y. Chen S. Zhang J. Liu B. (2023). Unravelling the complexity of lncRNAs in autophagy to improve potential cancer therapy. Biochimica Biophysica Acta Rev. Cancer 1878 (5), 188932. 10.1016/j.bbcan.2023.188932 37329993

[B163] Xiao H. Zhang L. Chen Y. Zhou C. Wang X. Wang D. (2020). PKM2 promotes breast cancer progression by regulating epithelial mesenchymal transition. Anal. Cell. Pathol. Amst. 2020, 8396023. 10.1155/2020/8396023 33294309 PMC7718057

[B164] Xiao M. Bian Q. Lao Y. Yi J. Sun X. Sun X. (2022). SENP3 loss promotes M2 macrophage polarization and breast cancer progression. Mol. Oncol. 16 (4), 1026–1044. 10.1002/1878-0261.12967 33932085 PMC8847990

[B165] Xiao Y. Yu D. (2021). Tumor microenvironment as a therapeutic target in cancer. Pharmacol. and Ther. 221, 107753. 10.1016/j.pharmthera.2020.107753 33259885 PMC8084948

[B166] Xing J. N. Shang Y. N. Yu Z. L. Zhou S. H. Chen W. Y. Wang L. H. (2024). LncRNA HCP5-encoded protein contributes to adriamycin resistance through ERK/mTOR pathway-mediated autophagy in breast cancer cells. Genes and Dis. 11 (4), 101024. 10.1016/j.gendis.2023.06.002 PMC10937560 38486678

[B167] Xu F. Guo M. Huang W. Feng L. Zhu J. Luo K. (2020). Annexin A5 regulates hepatic macrophage polarization via directly targeting PKM2 and ameliorates NASH. Redox Biol. 36, 101634. 10.1016/j.redox.2020.101634 32863213 PMC7369618

[B168] Xu M. Zhou C. Weng J. Chen Z. Zhou Q. Gao J. (2022). Tumor associated macrophages-derived exosomes facilitate hepatocellular carcinoma malignance by transferring lncMMPA to tumor cells and activating glycolysis pathway. J. Exp. and Clin. cancer Res. CR 41 (1), 253. 10.1186/s13046-022-02458-3 35986343 PMC9389814

[B169] Xu S. Wang P. Zhang J. Wu H. Sui S. Zhang J. (2019). Ai-lncRNA EGOT enhancing autophagy sensitizes paclitaxel cytotoxicity via upregulation of ITPR1 expression by RNA-RNA and RNA-protein interactions in human cancer. Mol. Cancer 18 (1), 89. 10.1186/s12943-019-1017-z 30999914 PMC6471868

[B170] Xue S. T. Zheng B. Cao S. Q. Ding J. C. Hu G. S. Liu W. (2022). Long non-coding RNA LINC00680 functions as a ceRNA to promote esophageal squamous cell carcinoma progression through the miR-423-5p/PAK6 axis. Mol. Cancer 21 (1), 69. 10.1186/s12943-022-01539-3 35255921 PMC8900330

[B171] Yang W. Xia Y. Hawke D. Li X. Liang J. Xing D. (2014). PKM2 phosphorylates histone H3 and promotes gene transcription and tumorigenesis. Cell 158 (5), 1210. 10.1016/j.cell.2014.08.003 28917293

[B172] Yang W. Xia Y. Ji H. Zheng Y. Liang J. Huang W. (2011). Nuclear PKM2 regulates β-catenin transactivation upon EGFR activation. Nature 480 (7375), 118–122. 10.1038/nature10598 22056988 PMC3235705

[B173] Yao R. W. Wang Y. Chen L. L. (2019). Cellular functions of long noncoding RNAs. Nat. Cell Biol. 21 (5), 542–551. 10.1038/s41556-019-0311-8 31048766

[B174] Yip C. W. Sivaraman D. M. Prabhu A. V. Shin J. W. (2021). Functional annotation of lncRNA in high-throughput screening. Essays Biochem. 65 (4), 761–773. 10.1042/EBC20200061 33835127 PMC8564734

[B175] Yofe I. Shami T. Cohen N. Landsberger T. Sheban F. Stoler-Barak L. (2023). Spatial and temporal mapping of breast cancer lung metastases identify TREM2 macrophages as regulators of the metastatic boundary. Cancer Discov. 13 (12), 2610–2631. 10.1158/2159-8290.CD-23-0299 37756565 PMC7617931

[B176] Yu Z. Wang C. Wang M. Li Z. Casimiro M. C. Liu M. (2008). A cyclin D1/microRNA 17/20 regulatory feedback loop in control of breast cancer cell proliferation. J. Cell Biol. 182 (3), 509–517. 10.1083/jcb.200801079 18695042 PMC2500136

[B177] Yu Z. Wang L. Wang C. Ju X. Wang M. Chen K. (2013). Cyclin D1 induction of Dicer governs microRNA processing and expression in breast cancer. Nat. Commun. 4, 2812. 10.1038/ncomms3812 24287487 PMC3874416

[B178] Zeng J. Hills S. A. Ozono E. Diffley J. F. X. (2023). Cyclin E-induced replicative stress drives p53-dependent whole-genome duplication. Cell 186 (3), 528–542.e14. 10.1016/j.cell.2022.12.036 36681079 PMC7619399

[B179] Zhang B. Shi J. Shi X. Xu X. Gao L. Li S. (2024a). Development and evaluation of a human CD47/HER2 bispecific antibody for Trastuzumab-resistant breast cancer immunotherapy. Drug Resist Updat. 74, 101068. 10.1016/j.drup.2024.101068 38402670

[B180] Zhang C. Wei S. Dai S. Li X. Wang H. Zhang H. (2023b). The NR_109/FUBP1/c-Myc axis regulates TAM polarization and remodels the tumor microenvironment to promote cancer development. J. Immunother. Cancer 11 (5), e006230. 10.1136/jitc-2022-006230 37217247 PMC10230994

[B181] Zhang Y. Wu M. J. Lu W. C. Li Y. C. Chang C. J. Yang J. Y. (2024b). Metabolic switch regulates lineage plasticity and induces synthetic lethality in triple-negative breast cancer. Cell Metab. 36 (1), 193–208.e8. 10.1016/j.cmet.2023.12.003 38171333

[B182] Zhang Z. Lu Y. X. Liu F. Sang L. Shi C. Xie S. (2023a). lncRNA BREA2 promotes metastasis by disrupting the WWP2-mediated ubiquitination of Notch1. Proc. Natl. Acad. Sci. U. S. A. 120 (8), e2206694120. 10.1073/pnas.2206694120 36795754 PMC9974429

[B183] Zhao M. Li J. Chen F. Han Y. Chen D. Hu H. (2023). Engineering nanoparticles boost TNBC therapy by CD24 blockade and mitochondrial dynamics regulation. J. Control. Release Official J. Control. Release Soc. 355, 211–227. 10.1016/j.jconrel.2023.01.075 36736908

[B184] Zhao Y. Yu Z. Ma R. Zhang Y. Zhao L. Yan Y. (2021). lncRNA-Xist/miR-101-3p/KLF6/C/EBPα axis promotes TAM polarization to regulate cancer cell proliferation and migration. Mol. Ther. Nucleic Acids 23, 536–551. 10.1016/j.omtn.2020.12.005 33510942 PMC7810606

[B185] Zheng F. Chen J. Zhang X. Wang Z. Chen J. Lin X. (2021). The HIF-1α antisense long non-coding RNA drives a positive feedback loop of HIF-1α mediated transactivation and glycolysis. Nat. Commun. 12 (1), 1341. 10.1038/s41467-021-21535-3 33637716 PMC7910558

[B186] Zhou L. Tian Y. Guo F. Yu B. Li J. Xu H. (2020). LincRNA-p21 knockdown reversed tumor-associated macrophages function by promoting MDM2 to antagonize* p53 activation and alleviate breast cancer development. Cancer Immunol. Immunother. CII. 69 (5), 835–846. 10.1007/s00262-020-02511-0 32062693 PMC11027865

[B187] Zhu S. Guo Y. Zhang X. Liu H. Yin M. Chen X. (2021). Pyruvate kinase M2 (PKM2) in cancer and cancer therapeutics. Cancer Lett. 503, 240–248. 10.1016/j.canlet.2020.11.018 33246091

[B188] Zhuang C. Liu Y. Fu S. Yuan C. Luo J. Huang X. (2020). Silencing of lncRNA MIR497HG via CRISPR/Cas13d induces bladder cancer progression through promoting the crosstalk between hippo/yap and TGF-β/smad signaling. Front. Mol. Biosci. 7, 616768. 10.3389/fmolb.2020.616768 33363213 PMC7755977

[B189] Zong S. Dai W. Guo X. Wang K. (2021). LncRNA-SNHG1 promotes macrophage M2-like polarization and contributes to breast cancer growth and metastasis. Aging 13 (19), 23169–23181. 10.18632/aging.203609 34618681 PMC8544328

